# Improved shrunken centroid classifiers for high-dimensional class-imbalanced data

**DOI:** 10.1186/1471-2105-14-64

**Published:** 2013-02-23

**Authors:** Rok Blagus, Lara Lusa

**Affiliations:** 1 Institute for Biostatistics and Medical Informatics, University of Ljubljana, Vrazov trg 2, Ljubljana, Slovenia

## Abstract

**Background:**

PAM, a nearest shrunken centroid method (NSC), is a popular classification method for high-dimensional data. ALP and AHP are NSC algorithms that were proposed to improve upon PAM. The NSC methods base their classification rules on shrunken centroids; in practice the amount of shrinkage is estimated minimizing the overall cross-validated (CV) error rate.

**Results:**

We show that when data are class-imbalanced the three NSC classifiers are biased towards the majority class. The bias is larger when the number of variables or class-imbalance is larger and/or the differences between classes are smaller. To diminish the class-imbalance problem of the NSC classifiers we propose to estimate the amount of shrinkage by maximizing the CV geometric mean of the class-specific predictive accuracies (g-means).

**Conclusions:**

The results obtained on simulated and real high-dimensional class-imbalanced data show that our approach outperforms the currently used strategy based on the minimization of the overall error rate when NSC classifiers are biased towards the majority class. The number of variables included in the NSC classifiers when using our approach is much smaller than with the original approach. This result is supported by experiments on simulated and real high-dimensional class-imbalanced data.

## Background

The objective of class prediction (classification) is to develop a rule based on variables measured on a group of samples with known class membership (training set), which can be used to assign the class membership to new samples (test set). Many different classifiers exist, and they differ in the definition of the classification rule [1]. Nowadays classification rules are increasingly often developed using data that are high-dimensional (the number of variables greatly exceeds the number of samples) and also class-imbalanced (the number of samples belonging to each class is not the same). High-dimensional classification has become a popular task in the biomedical and bioinformatics community with the advent of high-throughput technologies in biomedicine a decade ago. For example, many researchers attempted to develop gene-expression classifiers based on microarray experiments for prognostic and predictive purposes in breast cancer [2]. The number of subjects included in the microarray based classification studies is usually in the range of hundreds, while the number of measured genes is in the tens of thousands. Nowadays, the newly available next-generation sequencing methods provide billions of short reads for each subject, further increasing the high-dimensionality of data.

For high-dimensional data Tibshirani *et al.*[3] proposed the nearest shrunken centroid method (NSC, known also as prediction for microarrays - PAM), which can be seen as a modification of the diagonal linear discriminant analysis (DLDA [4]). The classification rule of DLDA is based on the scaled distance between the expression profiles of new samples and class centroids (vectors of class specific means); PAM uses a very similar rule, but it shrinks the class centroids towards the overall means and it embeds a variable selection mechanism, which is generally useful in high-dimensional class-prediction [4]. The amount of shrinkage is usually determined minimizing the cross-validated error rate on the training set [5,6]. Since its proposal, PAM has been widely used in practice. The paper that first described the PAM methodology [3] has been cited about thousand times, mostly in journals from the biomedical field: just papers from the fields of Oncology, Biochemistry and Biotechnology account for about half of the citations (source: ISI Web of Knowledge, accessed in November 2012).

The classifiers trained on class-imbalanced data tend to classify most of the new samples in the majority class [7] and this bias is further increased if data are high-dimensional [8]. It is somehow surprising that while DLDA can perform fairly well with imbalanced data (provided that the number of variables is reduced by some type of variable selection method) [4,8,9], PAM is very sensitive to the class-imbalance problem: it assigns most new samples to the majority class and achieves very poor accuracy for the minority class even when the level of class-imbalance is only moderate [8]. Even when the differences between classes are large the predictive accuracy tends to be smaller in the minority class. For example, Reeve and colleagues [10] used PAM to build a classifier to distinguish rejection from non-rejection kidney transplant using gene expression microarray data, achieving a better predictive accuracy for the majority class of non-rejection transplants: the cross-validated predictive accuracies were 80% (108 out of 135) in the non-rejection group and 69% in the rejection group (35 out of 51). Similarly, Korkola et al. [11] used PAM to predict the prognosis of 55 breast cancer patients and obtained a cross-validated predictive accuracy of 76% (26 out of 34) for good prognosis patients and of 62% (13 out of 21) for poor prognosis patients.

The class-imbalance bias of DLDA can be attributed to the larger variability of the estimate of the minority class centroid [8]; variable selection reduces the bias, but does not completely remove it if data are high-dimensional. The bias increases when the class-imbalance is larger and when more variables are measured because in these settings large discrepancies between sample values and true values are common in the minority class. Intuitively PAM should have an edge over DLDA in the class-imbalanced scenario, as shrinking the class centroids towards the overall centroid should reduce the extreme mean values that arise by chance and consequently diminish the class-imbalance bias.

Wang and Zhu [12] reinterpreted PAM in the framework of the LASSO regression [13] and proposed two classifiers that enable different amount of shrinkage for each variable (ALP: Adaptive *L*_*∞*_ norm penalized NSC and AHP: Adaptive hierarchically penalized NSC). They used simulated and real data to show that their methods outperform PAM in most circumstances, but did not address specifically the class-imbalance problem.

In this article we identify the features of the NSC classifiers that contribute to the class-imbalance bias and propose modified methods, GM-PAM, GM-ALP and GM-AHP, to reduce the class-imbalance bias. GM classifiers estimate the optimal shrinkage maximizing the cross-validated geometric mean of the class-specific predictive accuracies (g-means) and do not use the class prior correction that is embedded in the original classifiers.

The rest of the article is organized as follows. In the “Methods” section we present PAM, AHP and ALP classifiers. In section “Results” we first explain the pitfalls of the existing approach for the determination of the optimal threshold and present the novel algorithm; we apply the algorithm to three NSC classifiers and compare them with the existing approaches using simulated and real high-dimensional data. We end with a discussion and conclusions in Sections “Discussion” and “Conclusion”.

## Methods

Let *x*_*ij*_ be the value of variable *j* (*j*=1,…,*p*) for sample *i* (*i*=1,...,*n*). Each sample belongs to one of *K* classes (1,2,…,*K*) and *y*_*i*_ is the class of the *i*th sample. Let *z*_*ik*_ be a class membership indicator variable (*z*_*ik*_=1 if *y*_*i*_=*k*, *z*_*ik*_=0 otherwise), and $n_{k}=\overset{n}{\underset{i=1}{\sum }}z_{\text{ik}}$ be the number of samples in class *k*. The *j*th component of the centroid in class *k* is ${\bar{x}}_{\text{kj}}=\overset{n}{\underset{i=1}{\sum }}z_{\text{ik}}x_{\text{ij}}/n_{k}$ and the *j*th component of the overall centroid is ${\bar{x}}_{j}=\overset{n}{\underset{i=1}{\sum }}x_{\text{ij}}/n$. The shrunken centroid is defined as

(1) $${\bar{x}}_{\text{kj}}^{,}={\bar{x}}_{j}+{\hat{d}}_{\text{kj}}\cdot m_{k}\cdot (s_{j}+s_{0}),$$

where *s*_*j*_ is the pooled within-class standard deviation for the *j*th variable, *s*_0_ is a constant (set at the median of *s*_*j*_), $m_{k}=\sqrt{1/n_{k}-1/n}$ and in PAM ${\hat{d}}_{\text{kj}}$ is defined as

(2) $${\hat{d}}_{\text{kj}}=\text{sgn}(d_{\text{kj}}){\left(|d_{\text{kj}}|-\lambda \right)}_{+},$$

where $d_{\text{kj}}=\frac{{\bar{x}}_{\text{kj}}-{\bar{x}}_{j}}{m_{k}(s_{j}+s_{0})}$, *λ*≥0 is a threshold parameter that needs to be tuned, and (·)_+_ is the positive part of (·).

The classification rule of PAM for a new sample **x**^∗^ is

(3) $$C(x^{*})={\text{argmin}}_{k}\delta_{k}(x^{*}),$$

where *δ*_*k*_(**x**^∗^) is the discriminant score for class *k*, defined as

(4) $$\delta_{k}(x^{*})=\overset{p}{\underset{j=1}{\sum }}\frac{{\left(x_{j}^{*}-{\bar{x}}_{\text{kj}}^{,}\right)}^{2}}{{\left(s_{j}+s_{o}\right)}^{2}}-2\log(\pi_{k})=\overset{p}{\underset{j=1}{\sum }}L_{\text{kj}}-2\log(\pi_{k});$$

*π*_*k*_ is the proportion of class *k* samples in the population ($\overset{K}{\underset{k=1}{\sum }}\pi_{k}=1$), −2*log*(*π*_*k*_) is a class prior correction and $L_{k}=\overset{p}{\underset{j=1}{\sum }}L_{\text{kj}}$.

Variable *j* is effectively not considered in the classification rule (inactive variable) when all ${\bar{x}}_{\text{kj}}^{,}$ are shrunken to ${\bar{x}}_{j}$ as $L_{1j}=\cdots =L_{\text{Kj}}$; we call the other variables active.

Wang and Zhu [12] showed that if the observation *x*_*i*_=(*x*_*i*1_,...,*x*_*ip*_) from class *k* follows a multivariate normal distribution (*MVN*(**μ**_*k*_,**Σ**_*k*_)) and the covariance matrices are the same across different classes and are diagonal ($\Sigma_{k}=\text{diag}(\sigma_{1}^{2},\mathrm{...},\sigma_{p}^{2})$) then (2) is a solution to

(5) $${\hat{d}}_{\text{kj}}={\text{argmin}}_{{\tilde{d}}_{\text{kj}}}\frac{1}{2}\overset{n}{\underset{i=1}{\sum }}\overset{p}{\underset{j=1}{\sum }}\overset{K}{\underset{k=1}{\sum }}\frac{z_{\text{ik}}}{n_{k}}{\left({\tilde{x}}_{\text{ij}}-{\tilde{d}}_{\text{kj}}\right)}^{2}+\lambda \;\overset{p}{\underset{j=1}{\sum }}\overset{K}{\underset{k=1}{\sum }}|{\tilde{d}}_{\text{kj}}|$$

where ${\tilde{x}}_{\text{ij}}=\frac{x_{\text{ij}}-{\bar{x}}_{j}}{m_{k}(s_{j}+s_{0})}$, ${\tilde{d}}_{\text{kj}}=\frac{\mu_{\text{kj}}-{\bar{x}}_{j}}{m_{k}(s_{j}+s_{0})}$ and $\overset{p}{\underset{j=1}{\sum }}\overset{K}{\underset{k=1}{\sum }}|{\tilde{d}}_{\text{kj}}|$ is a penalty function. Based on the observation that (5) is a LASSO type estimator for ${\hat{d}}_{\text{kj}}$, Wang and Zhu [12] proposed two different penalty functions,

(6) $$\;\;\;\;\;\lambda \overset{p}{\underset{j=1}{\sum }}w_{j}\cdot \underset{k}{\max}(|{\tilde{d}}_{1j},\mathrm{...},{\tilde{d}}_{\text{Kj}}|)\text{, and}$$

(7) $$\;\;\;\;\;\lambda_{\gamma }\overset{p}{\underset{j=1}{\sum }}w_{j}^{\gamma }\gamma_{j}+\lambda_{\theta }\overset{p}{\underset{j=1}{\sum }}\overset{K}{\underset{k=1}{\sum }}w_{\text{kj}}^{\theta }|\theta_{\text{kj}}|,$$

where *w*_*j*_, $w_{j}^{\gamma }$ and $w_{\text{kj}}^{\theta }$ are pre-specified weights and *λ*, *λ*_*γ*_ and *λ*_*θ*_ are threshold parameters (see Additional file 1 for the definition of *γ*_*j*_ and *θ*_*kj*_).

The shrunken centroids, discriminant scores and classification rules are the same as in PAM; the classification rules that use (6) and (7) are denoted with ALP and AHP, respectively.

PAM, ALP and AHP require the estimation of the threshold parameter *λ*, *λ*_*γ*_ and *λ*_*θ*_. A normal procedure is to use the training data to estimate a cross-validated (CV) error rate for different values of the threshold and use the threshold that produces the lowest overall error [5]. Note that when the threshold is zero, then the classification rules of PAM, ALP and AHP are essentially the same as the classification rule of DLDA (with the exception of an added constant *s*_0_ that is not considered in DLDA), which is defined as

(8) $$\;\;\;\;\;\delta_{k}(x^{*})=\overset{p}{\underset{j=1}{\sum }}\frac{{\left(x_{j}^{*}-{\bar{x}}_{\text{kj}}\right)}^{2}}{s_{j}^{2}}-2\log(\pi_{k})=L_{k}-2\log(\pi_{k}),$$

where *L*_*k*_ is the discriminant score omitting the class prior correction.

In practice for high dimensional data the class prior correction contributes little to the discriminant scores (|*L*_*k*_|>>−2 log(*π*_*k*_) and *δ*_*k*_≈*L*_*k*_ for large *p*), while it can bias the NSC classification towards the majority class if all or most of the variables are inactive (*L*_*k*_≈0 and *δ*_*k*_≈−2 log(*π*_*k*_)). For these reasons we used equal class priors for all the classes (−2 log(1/*K*)), similarly as Huang *et al.*[14]. Moreover, in case of ties the class membership was assigned at random to one of the classes with the smallest discriminant scores.

## Results

In this section we discuss the implications of estimating the optimal threshold for NSC classifiers by minimizing the cross-validated overall error, when data are class-imbalanced and high-dimensional. We then present a modified approach for threshold estimation aimed at reducing the class-imbalance problem for NSC classifiers, and show its effectiveness on simulated and real high dimensional class-imbalanced data.

### Threshold selection

In practice the threshold parameters of the NSC classifiers are estimated minimizing the cross-validated error rate for different values of the threshold; the threshold value that produces the lowest error is used to shrink the centroids.

The overall error is the probability of misclassifying new samples:

(9) $$\text{error}=1-\overset{K}{\underset{k=1}{\sum }}P(C(x^{*})=k|y^{*}=k)\pi_{k},$$

and it depends on the class specific predictive accuracies (${\text{PA}}_{k}=P(C(x^{*})=k|y^{*}=k)$) and on the level of class imbalance. Overall error and predictive accuracy (1-error) are misleading measures of the classifiers performance when data are class-imbalanced [15]: the predictive accuracies of the minority classes are given little weight and classifying all new samples in the majority class produces small overall error when the class-imbalance is extreme.

**Figure 1:**
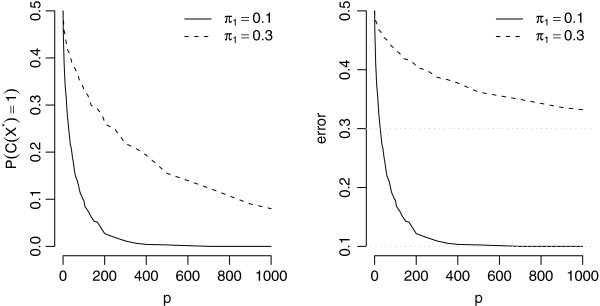
**Probability of classification of a new sample in the minority class and the classification error as a function of the number of variables.** The figure shows the probability of classification of a new sample in the minority class (left panel) and the classification error (right panel) as a function of the number of variables for the example presented in the main text.

The high-dimensionality of data can additionally contribute to making the overall error an inappropriate measure to minimize. For the sake of simplicity let us focus on DLDA; we consider a two-class classification problem and assume that there is no real difference between the classes (null case) and that class 1 is the minority class (known *π*_1_<0.5). Data are simulated from *x*_*i*_∼iid*MVN*(**0**,**Σ**=*diag*(1,...,1)) for both classes for *n*=100 samples, with *π*_1_=0.10 or *π*_1_=0.30; results are shown in Figure 1.

The probability of classifying a new sample in the minority class is smaller when the class imbalance is more extreme and/or when more variables are measured. As a consequence, the error rate is a decreasing function of the number of variables and, when the number of variables is large, it approaches the proportion of the minority class samples in the population. For this particular setting the classification probabilities were derived also analytically, additionally assuming that the variances are known, see Additional file 2.

If we consider the shrunken centroid classifiers as a special form of DLDA this result would suggest that in the class-imbalanced scenario the threshold selection based on the overall error will favor small threshold values (large number of variables), which in turn will lead to small probability of classification in the minority class and large bias in favor of the majority class.

### The proposed approach

We propose to select the optimal threshold as the value that maximizes the cross-validated geometric mean of the class specific predictive accuracies (GM, g-means),

(10) $$\text{GM}=\sqrt[{K}]{\overset{K}{\underset{k=1}{\prod }}{\text{PA}}_{k}}.$$

g-means is an accuracy metric often used for class-imbalanced data that captures the performance of the classifiers in all classes [7]. It gives the same weight to all the classes, it is independent of the class distribution of the test set and it penalizes the classifiers whose performance is heterogeneous across classes. Furthermore, for a fixed total ($\overset{K}{\underset{k=1}{\sum }}PA_{k}$), it has the maximum when the class specific predictive accuracies are equal [16].

In practice the class specific PA are estimated with ${\text{PA}}_{k}=1/n_{k}\overset{n}{\underset{i=1}{\sum }}z_{\text{ik}}\cdot z_{i\hat{y}}$, where $z_{i\hat{y}}$ is the indicator for a correctly classified sample *i* ($z_{i\hat{y}}=1$ if $C(x_{i})=y_{i}$ and zero otherwise) and they depend on the selected threshold value. It is not feasible to evaluate the cross-validated GM for all possible threshold values, therefore we limit our attention to a fixed number of thresholds. We consider *T* equally spaced threshold values, ranging from 0 (no shrinkage) to *λ*_max_, the minimum threshold value that shrinks all the class centroids to the overall centroid, for all the variables (complete shrinkage). In the Additional file 1 we show how to derive *λ*_max_ for PAM, ALP and AHP.

The proposed approach for the estimation of the threshold can be used for each of the three NSC classifiers considered in this paper; the modified classifiers are denoted with GM-PAM, GM-ALP and GM-AHP, respectively. The proposed algorithm is presented below.

**Algorithm 1** GM shrunken centroid classifier

## Results on the simulated data

In this section we present a series of selected results based on simulated data to assess the performance of the GM method and compare it with the original NSC classifiers.

In a two class classification scenario, we simulated 10,000 variables from a multivariate Gaussian distribution. We used a block exchangeable correlation structure, in which the variables in the same block were correlated (pairwise correlation equal to *ρ*=0.8) while the variables from different blocks were independent (similarly as in Guo *et al.*[17] and Pang *et al.*[18]); each block contained 100 variables and all variances were equal to 1. The mean values were equal to 0 for all variables in class 1 (*μ*_1_=**0**). In the null case all the variables were non informative (*μ*_1_=*μ*_2_=**0**), while in the alternative case 100 variables were informative about class distinction (*μ*_2_=0.5, 1, 2 or 5 for the informative variables and *μ*_2_=0 for non informative variables).

The training sets contained 100 samples and the proportion of class 1 samples varied from 0.5 (balanced situation) to 0.9 (highly imbalanced situation). 10-fold CV was used to estimate the optimal threshold parameter, using 30 different threshold values. The classifier trained on the complete data set with the estimated optimal threshold was used to make predictions on a large independent and balanced test set (*n*_*test*_=1,000, $k_{1}^{\text{test}}=0.5$, where $k_{1}^{\text{test}}$ is the proportion of class 1 samples in the test set), simulated from the same distribution used for the training set. The performance on the test set was evaluated in terms of class-specific predictive accuracies, g-means, the area under the ROC curve (AUC): these measures do not depend on the data distribution in the test set and can be estimated with equal precision when the test set classes are balanced. Furthermore, it was previously shown that matching the prevalence in the training and test set does not attenuate the class-imbalance problem [8]. In the simulations we evaluated also the false discovery rate (FDR, proportion of non informative variables among active variables) and the false negative rate (FNR, proportion of informative variables among the non active variables). Each simulation was repeated 500 times.

**Figure 2:**
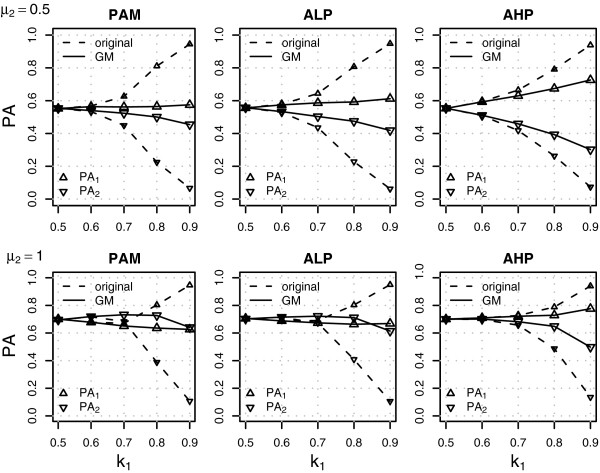
**Classification results under the alternative hypothesis for the NSC and GM-NSC classifiers.** The figure shows class specific predictive accuracies (PA_1_ and PA_2_) for different levels of class-imbalance (*k*_1_) in the training set. The differences between the classes were small (upper panel: *μ*_2_=0.5) or moderate (lower panel: *μ*_2_=1). See text for details.

The NSC classifiers assigned most new samples to the majority class when there was no difference between the classes (Additional files 3 and 4) or the difference between classes was small or moderate (Figure 2 and Table 1). The bias towards the majority class was smaller when the classes were more balanced, when the number of variables was smaller (the results of additional simulations using 1000 variables are presented in the Additional files 5, 6 and 7) or the difference between classes was larger. The NSC classifiers were not effective in removing the non-informative variables: the number of active variables markedly increased with class-imbalance and most of the variables were not shrunken towards the overall centroid in the most imbalanced settings; as a consequence the FDR was close to 1. In general, AHP had less active variables and a slightly smaller FDR, but the overall performance of PAM, ALP and AHP was similar.

**Table 1:** **Performance of the classifiers under the alternative hypothesis with large class-imbalance (*k***_***1***_*** = 0.9*) and moderate differences between classes (*μ***_***2***_*** =1 *)**

**Method**	***λ***^***∗***^^**a**^	**#, % info**	**# non-info [%]**	**FDR**	***PA***_***1***_	***PA***_***2***_	**g-means**	**AUC**
					***(n***_***1***_*** = 90)***	***(n***_***2***_*** = 10)***		
PAM	0.05	99.94	9184.1 [92.77]	0.99	0.95	0.11	0.31	0.6
	(0.08)	(0.87)	(1136.28)	(0.00)	(0.02)	(0.05)	(0.07)	(0.04)
GM-PAM	1.29	58.81	602.1 [6.08]	0.62	0.63	0.64	0.62	0.69
	(0.51)	(43.6)	(1004.73)	(0.38)	(0.1)	(0.16)	(0.08)	(0.1)
ALP	0.07	99.96	9004.2 [90.95]	0.99	0.95	0.11	0.3	0.61
	(0.19)	(0.54)	(2232.18)	(0.01)	(0.03)	(0.07)	(0.08)	(0.04)
GM-ALP	3.76	63.08	408.8 [4.13]	0.59	0.67	0.61	0.63	0.68
	(2.21)	(41.09)	(816.3)	(0.36)	(0.1)	(0.17)	(0.09)	(0.09)
AHP	0.36	96.89	6438.9 [65.04]	0.95	0.94	0.14	0.34	0.62
	(1.57)	(12.84)	(4235.52)	(0.11)	(0.04)	(0.1)	(0.1)	(0.05)
GM-AHP	5.29	37.45	266.5 [2.69]	0.42	0.78	0.5	0.6	0.69
	(3.94)	(37.96)	(1275.07)	(0.4)	(0.08)	(0.18)	(0.12)	(0.1)

The table reports the estimated optimal threshold (*λ*^∗^), the number [%] of active non-informative variables (# non-info [%], selected out of 9,900 non-informative variables) and the number (also equal to %) of active informative variables (#, % info, selected out of 100 informative variables, equal to 100(1-false negative rate)), false discovery rate (FDR, # non-info/(# active)), class specific predictive accuracies, g-means and AUC, averaged over 500 repetitions; standard deviations are reported in brackets. The simulation settings are the same as in Figure 2. ^a^ For AHP and GM-AHP only *λ*_*θ*_ was optimized while *λ*_*γ*_ was set to zero.

The GM-NSC classifiers performed very similarly to NSC classifiers in the settings where the NSC classifiers were not biased towards the majority class, i.e. when the classes were balanced or were very different (data not shown). In the other situations the GM-NSC classifiers reduced the gap between the class specific PA, obtaining larger minority class PA, g-means and AUC, and greatly reducing the number of active variables; the removal of most of the non informative variables reduced the FDR and the bias towards the classification into the majority class (Table 1), while the removal of a part of the informative variables increased the false negative rate (FNR). The best performance was obtained when the GM threshold optimization was used with PAM, while the smallest improvement was seen for AHP. This can probably be attributed to the fact that the variables with larger *d*_*kj*_ values are weighted and therefore shrunken less, which is not desirable for the non informative variables as large values of *d*_*kj*_ arose by chance and should therefore be actually shrunken more; note that the smallest bias was observed for PAM (with GM approach), where all variables are shrunken for the same amount. Similar results were obtained simulating independent variables (data not shown).

When some variables were differentially expressed and the class-imbalance was moderate, methods using the GM approach achieved slightly higher PA for the minority class, while this bias was only marginal if the original approach was used. Note that the overall centroid can be expressed as ${\bar{x}}_{j}=\overset{K}{\underset{k=1}{\sum }}{\bar{x}}_{\text{kj}}n_{k}/n$; it is a weighted average of class specific mean values with more weight given to the majority class, so the overall centroid is closer to the sample mean of the majority class. When the threshold is large this has a consequence of shifting the minority class centroid towards the sample mean of the majority class and hence some of the new samples from the majority class are closer to the minority class (shrunken) centroid than to the majority class (shrunken) centroid. Classifiers using the original approach do not suffer from this problem as the amount of shrinkage is small when the training set is class-imbalanced. One way of diminishing this bias would be to define the overall centroid as ${\bar{x}}_{j}=\overset{K}{\underset{k=1}{\sum }}{\bar{x}}_{\text{kj}}/K$, and $m_{k}=\sqrt{\frac{1}{K}\left(\frac{K-2}{n_{k}}+\frac{1}{K}\overset{K}{\underset{k=1}{\sum }}n_{k}^{-1}\right)}$ as to assure that the denominator in calculation of *d*_*kj*_ will be the appropriate standard error. We performed a limited set of simulations with this overall centroid definition for PAM and observed that the bias in favor of the minority class was removed. However, when there was large class-imbalance, the results were slightly more biased in favor of the majority class (data not shown) than with the original overall centroid definition. One reason for poor performance in the case of large class-imbalance is that relatively more weight was given to a less accurate estimate.

One of the possible strategies when dealing with class-imbalanced data is to use case weighting in order to adjust for the class-imbalance bias [7]. Since case weighting is not implemented in the NSC classifiers we performed a limited set of experiments with random over-sampling to give the same weight to both classes. Class balanced training sets were obtained by replicating a subset of randomly selected samples from the minority class (replicating max(*n*_1_,*n*_2_)− min(*n*_1_,*n*_2_) samples from the minority class and obtaining the training set of size 2 max(*n*_1_,*n*_2_)). PAM and GM-PAM were trained on the over-sampled training sets and evaluated on independent test sets. The simulation settings and the settings of PAM and GM-PAM were the same as presented above (see Additional file 8 for the results).

Over-sampling did not significantly change the performance of PAM, while it increased the class imbalance bias of GM-PAM. After over-sampling PAM and GM-PAM performed exactly the same, achieving poor predictive accuracy for the minority class when the original training set was class-imbalanced. The number of active variables in GM-PAM was much larger than in the simulations where over-sampling was not used. After over-sampling the training set contains exact copies of the minority class samples. When the level of class-imbalance is severe there are many copies of the same minority class sample in the training set and the predictive accuracy for the minority class obtained by using cross-validation is (nearly) a re-substitution (training) estimate, as the same minority class samples can be used in the training and testing phase. The fact that the GM-PAM approach favored the use of large number of variables can be explained by realizing that classifiers that use many variables minimize the re-substitution PA (in our case minority class PA) [19,20]; because of the class-imbalance bias the majority class PA is also increasing when using more variables. Consequently, the g-means is an increasing function of the the number of variables, and is maximized when the amount of shrinkage is small. The use of large number of variables (small threshold) translates into a large class-imbalance bias on the independent test set.

We considered also a three class scenario, simulating 5,000 variables from a multivariate Gaussian distribution; the correlation structure was the same as in the two-class scenario. We considered the null case and the alternative case where class 2 was the minority class nested between class 1 and class 3 (*μ*_1_=−*μ*_3_=1 and *μ*_2_=0 for 100 informative variables, and *μ*_1_=*μ*_2_=*μ*_3_=0 for non informative variables; *n*_2_=20, *n*_1_=*n*_3_=100). The test sets were balanced (*n*_*test*_=1500) and the classifiers were trained and evaluated as described for the two-class scenario. The null case results are in the Additional file 9, where additional simulation results with 1000 variables and balanced data (*n*_1_=*n*_2_=*n*_3_=100) are also presented.

In the null case the NSC classifiers assigned most new samples to the majority classes and the proportion of samples classified in the minority class decreased when more variables were considered. The GM-NSC classifiers assigned approximately the same number of samples to each class. All the classifiers performed similarly on balanced data, classifying approximately the same number of samples to each class. In the alternative case the NSC classifiers obtained very low PA for the minority class and PAM performed worse than ALP and AHP (Table 2). The GM-NSC classifiers performed better, substantially increasing the minority class PA and g-means, in spite of using a larger number of non-informative variables. Note that class 2 is the hardest class to predict also with balanced data (see Additional file 9) and that the GM-NSC classifiers achieved approximately the same classification results on balanced and imbalanced data, showing to be insensitive to class imbalance in this setting.

**Table 2:** Multi-class classification results for the class-imbalanced scenario in the alternative case

**Method**	***λ***^***∗***^^**a**^	**#, % info**	**# non-info [%]**	**FDR**	***PA***_***1***_	***PA***_***2***_	***PA***_***3***_	**g-means**
					***(n***_***1***_*** = 100)***	***(n***_***1***_*** = 20)***	***(n***_***3***_*** = 100)***	
PAM	6.6	59.23	0 [0.00]	0	0.86	0.04	0.86	0.29
	(0.58)	(33.68)	(0)	(0.00)	(0.02)	(0.02)	(0.02)	(0.05)
GM-PAM	1.4	99.64	834.56 [17.03]	0.5	0.75	0.31	0.75	0.55
	(0.96)	(5.75)	(1262.68)	(0.41)	(0.04)	(0.06)	(0.04)	(0.04)
ALP	58.76	99.98	49 [1.00]	0.01	0.82	0.15	0.82	0.46
	(15.12)	(0.14)	(490)	(0.1)	(0.04)	(0.05)	(0.03)	(0.04)
GM-ALP	12.77	99.64	415.68 [8.48]	0.19	0.74	0.34	0.74	0.57
	(12.88)	(3.6)	(1330.05)	(0.31)	(0.05)	(0.08)	(0.05)	(0.05)
AHP	59.05	99.99	98 [2.00]	0.02	0.82	0.15	0.82	0.45
	(15.74)	(0.1)	(689.46)	(0.14)	(0.04)	(0.05)	(0.04)	(0.05)
GM-AHP	13.55	100	316.73 [6.46]	0.17	0.74	0.34	0.74	0.57
	(13.05)	(0)	(1164.99)	(0.28)	(0.05)	(0.08)	(0.05)	(0.04)

The table reports the same information as Table 1; # non-info [%] was selected out of 4,900 non-informative variables and #, % info was selected out of 100 informative variables; see text for details.

^a^For AHP and GM-AHP only *λ*_*θ*_ was optimized while *λ*_*γ*_ was set to zero.

## Application to real high-dimensional data sets

We used four breast cancer microarray gene expression data to assess the performance of the GM-NSC and NSC classifiers. We predicted the Estrogen receptor (ER) positivity, the histological grade (Grade 1 and 2 vs Grade 3, or as a three class prediction problem), the disease relapse and the prognosis of the breast cancer patients (good or bad). Grade, prognosis and relapse are harder to predict than ER status using breast cancer gene expression data [21]. Table 3 summarizes the main characteristics of the data sets originally published by Ivshina *et al.*[22], Wang *et al.*[23], Sotiriou *et al.*[21] and Korkola *et al.*[11], and the classification tasks addressed. We used 5-fold CV to estimate the optimal threshold parameter and, if not noted otherwise, the accuracy measures were estimated using leave-one-out CV (LOOCV).

**Table 3:** Gene expression breast cancer data sets

**Data set**	**# genes**	**Classification task**	***n***_**1**_	***n***_**2**_	***n***_**3**_	***k***_***m i n***_^**a**^
Ivshina	22,283	ER- or ER+	34	211		0.14
		Grade 1, 2 or 3	68	166	55	0.19
		Grade 1, 2 or 3	40	10 to 80	40	0.25 to 0.50
Wang	22,283	Relapse or not	179	107		0.37
Korkola	9,524	Good or bad prognosis	34	21		0.38
Sotiriou	7,650	ER+ or ER-	10 to 50	10		0.50 to 0.17
		Grade 1-2 or 3	10 to 40	10		0.50 to 0.20

^a^***k***_***min***_ is a proportion of the minority class samples in the training set.

All the classifiers performed well in predicting the ER status on the Ivshina data set (Table 4). AHP used few active genes and had the best performance among the NSC classifiers, while PAM and ALP did not remove any of the genes nor did they shrink any of the components of the centroids. The gap between the class specific PA was small despite the large class imbalance, because the difference between the classes was large. Nevertheless, the GM classifiers used fewer active genes and improved the minority class PA, g-means and AUC of PAM and ALP, and performed similarly to AHP. The absence of shrinkage for PAM and ALP in the prediction of ER status on the Ivshina’s data set can be attributed to the large class imbalance (*k*_ER-_=0.14) and to the fact that, unless there was very little or no shrinkage, the minority (ER-) class had better class specific PA in this data set. The bias towards classification into the majority class, caused by the use of many non informative variables, appeared only when most or all the variables were included in the classifier (see Additional file 10). The majority class PA and consequently the overall PA were maximized by the classifier with no shrinkage.

**Table 4:** Performance of the classifiers on real gene expression data sets for the two class classification tasks

**Data set**	**Method**	***λ***^**∗**^^**a**^	**# genes**	**PA**	**PA**_**1**_	**PA**_**2**_	**g-means**	**AUC**
Ivshina	PAM	0	22283	0.84	0.79	0.85	0.82	0.85
(ER)	GM-PAM	4.83	51	0.82	0.91	0.81	0.86	0.90
	ALP	0	22283	0.84	0.82	0.84	0.83	0.86
	GM-ALP	58.24	26	0.85	0.91	0.84	0.87	0.88
	AHP	185.56	20	0.89	0.88	0.89	0.88	0.90
	GM-AHP	69.58	115	0.89	0.88	0.89	0.89	0.91
Wang	PAM	3.71	14	0.61	0.60	0.62	0.61	0.62
	GM-PAM	3.71	14	0.60	0.60	0.62	0.61	0.63
	ALP	8.26	654	0.56	0.57	0.55	0.56	0.63
	GM-ALP	8.26	654	0.56	0.56	0.56	0.56	0.63
	AHP	21.95	135	0.56	0.56	0.56	0.56	0.65
	GM-AHP	21.95	135	0.56	0.56	0.55	0.56	0.63
Korkola	PAM	0.19	7073	0.65	0.71	0.57	0.64	0.64
	GM-PAM	0.19	7073	0.65	0.71	0.57	0.64	0.64
	ALP	4.87	155	0.64	0.65	0.62	0.63	0.64
	GM-ALP	4.87	155	0.69	0.74	0.62	0.67	0.69
	AHP	0.76	1308	0.58	0.68	0.43	0.54	0.58
	GM-AHP	0.76	1308	0.62	0.71	0.48	0.58	0.60

The table reports the same information as Table 1; # genes is the number of active genes. Optimal thresholds were estimated with 5-fold CV and the accuracy measures with LOOCV; see text for details.

^a^For AHP and GM-AHP only *λ*_*θ*_ was optimized while *λ*_*γ*_ was set to zero.

Relapse was more difficult to predict on Wang’s data set (Table 4). PAM used the smallest number of active genes and performed slightly better than ALP and AHP in terms of class specific PA and g-means. Exactly the same results were obtained using NSC and GM-NSC classifiers. This result is in line with the simulations, where we showed that for moderate class-imbalance the performance of NSC and GM-NSC classifiers was very similar.

PAM used the largest number of variables on Korkola’s data set (Table 4) but still performed better than AHP and ALP that used less variables. The performance of GM-PAM and GM-AHP was similar to the original methods, while GM-ALP outperformed ALP and achieved the best overall performance on this data set.

In order to explore the effect of class-imbalance on real data, we obtained multiple training sets from the Sotiriou’s data set, varying the level of class-imbalance. We used a fixed number of samples from the minority class (*n*_ER-_=10) and varied the number of samples from the majority class (*n*_ER+_=10,20,…,50); the samples not included in the training set were used to estimate the accuracy measures. To account for the variability arising from random inclusion of samples in the training or test set, we repeated the procedure 250 times and averaged the results.

**Figure 3:**
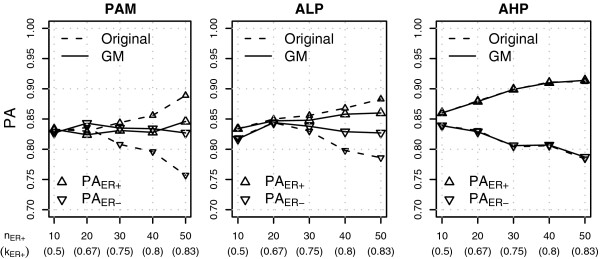
**Classification results on the Sotiriou data set.** The figure shows PA for ER+ class (PA_ER+_) and PA for ER- class (PA_ER-_) for different number of ER+ samples in the training set (*n*_ER+_). There were 10 ER- samples in each training set. See text for more details.

In the balanced situation the class specific PA were approximately equal for all classifiers, indicating that the classes were roughly equally difficult to predict (Figure 3, see Additional file 11 for exact numerical results). For the NSC classifiers the PA of the minority class decreased when majority class samples were added to the training set, while the majority class PA increased, similarly as observed in our simulations. Moreover, the number of active genes increased substantially, especially for PAM and ALP. GM-PAM and GM-ALP were effective in maintaining the minority class PA above the values achieved on class-balanced data and the gap between the class specific PA was very small even when the class-imbalance was large. The number of active genes increased with class-imbalance for GM-PAM and GM-ALP but not as dramatically as for the original methods. GM-AHP performed very similarly to AHP. Comparable results were obtained for the two-class prediction of grade (Grade 1 or 2 vs Grade 3; Additional file 11).

We addressed also a three-class classification problem on Ivshina’s data, predicting the grade of tumors (1, 2 or 3). On the complete data set all the classifiers performed very similarly (Additional file 11). Only GM-PAM removed a part of the variables, while the other classifiers did not shrink the centroids at all. Grade 2 was the majority class but it had the worst PA, indicating that the potentially heterogeneous Grade 2 class is the most difficult to predict. Similarly as on the Sotiriou’s data, we varied the number of samples in Grade 2 class to try to isolate the class-imbalance effect (*n*_Grade 1_=*n*_Grade 3_=40 and *n*_Grade2_=10,20,40 or 80). When Grade 2 was the majority class the results were very similar to those obtained on the complete data set, and GM-NSC and NSC classifiers performed similarly (Table 5, *n*_2_=80). In the balanced setting Grade 2 had the lowest PA, confirming that Grade 2 was the most difficult class to predict; GM improved the performance of PAM and ALP.

**Table 5:** Results on the Ivshina data set for classification of Grade of the tumor

***n***_***Grade 2***_	**Method**	***λ***^**∗**^^**a**^	**# genes**	**PA**	**PA**_**1**_	**PA**_**2**_	**PA**_**3**_	**g-means**
10	PAM	4.25	963	0.29	0.90	0.12	0.89	0.45
	GM-PAM	2.76	5768	0.34	0.86	0.20	0.88	0.51
	ALP	10.26	7425	0.33	0.88	0.18	0.88	0.51
	GM-ALP	28.39	3295	0.37	0.70	0.26	0.88	0.50
	AHP	25.61	9508	0.39	0.83	0.27	0.85	0.57
	GM-AHP	76.40	640	0.45	0.75	0.36	0.86	0.61
20	PAM	3.14	3023	0.34	0.89	0.18	0.88	0.51
	GM-PAM	1.27	12299	0.42	0.83	0.30	0.85	0.59
	ALP	11.20	5541	0.40	0.85	0.26	0.88	0.57
	GM-ALP	10.67	9991	0.42	0.81	0.30	0.86	0.58
	AHP	30.66	7733	0.47	0.78	0.37	0.83	0.62
	GM-AHP	48.52	3811	0.48	0.76	0.39	0.83	0.62
40	PAM	1.55	9832	0.46	0.86	0.32	0.87	0.61
	GM-PAM	0.49	17439	0.52	0.83	0.41	0.84	0.65
	ALP	12.43	6885	0.45	0.82	0.31	0.87	0.59
	GM-ALP	2.00	16919	0.51	0.80	0.41	0.83	0.64
	AHP	37.43	6366	0.52	0.73	0.44	0.83	0.64
	GM-AHP	32.21	7148	0.53	0.74	0.44	0.82	0.64
80	PAM	0.28	19617	0.58	0.77	0.48	0.83	0.67
	GM-PAM	0.32	19311	0.58	0.78	0.47	0.83	0.67
	ALP	0.68	19149	0.59	0.75	0.50	0.82	0.67
	GM-ALP	0.76	18727	0.59	0.75	0.50	0.82	0.67
	AHP	15.20	11231	0.58	0.73	0.48	0.82	0.66
	GM-AHP	14.69	10928	0.57	0.73	0.48	0.82	0.66

The table reports the same information as Table 4. There were 40 Grade 1 and Grade 3 samples and the number of Grade 2 samples varied; see text for more details.

^a^For AHP and GM-AHP only *λ*_*θ*_ was optimized while *λ*_*γ*_ was set to zero.

Decreasing the number of Grade 2 samples had the effect of further decreasing the PA of Grade 2 and the g-means; the PA of the other two classes, which were high when data were balanced, increased only moderately for most classifiers. The drop in the PA of Grade 2 was less pronounced for the GM classifiers. Similarly as for the other classification tasks the GM method was the most useful in improving the performance of PAM and ALP.

## Discussion

In this paper we proposed a modified approach (GM-NSC) to the estimation of the amount of centroid shrinkage for the NSC classifiers. The approach estimates the optimal shrinkage by maximizing the geometric mean of the class-specific predictive accuracies, rather than the overall accuracy. We used our approach with PAM and with two recently proposed NSC classifiers, ALP and AHP.

The motivation for the new approach is to alleviate the class-imbalance problem of the NSC classifiers. We showed with a limited set of simulations that ALP and AHP, similarly to PAM [8], are biased towards the classification in the majority class when data are class-imbalanced: they assign most new samples to the majority class and achieve poor predictive accuracy for the minority class, unless the differences between the classes are very large. Increasing the number of measured variables has the effect of further increasing the bias.

We identified the main reason for the biased NSC classification in the method used in practice for estimating the threshold parameter, which is based on the minimization of the cross-validated overall error rate. The threshold parameter plays a fundamental role for NSC classifiers, as it determines how many variables are effectively used in the classification rule and by which amount the centroids are shrunken.

Simulation results and the analysis of three large data sets of breast cancer showed that the greatest gains were obtained by GM-NSC when the NSC classifiers had a large bias towards the majority class, while GM-NSC performed similarly to NSC in the absence of bias. GM-NSC classifiers used less active variables when data were class-imbalanced.

In the biomedical applications the improvements obtained using the GM-NSC classifiers are relevant from the practical point of view. The reduction of the class-imbalance bias results in more accurate prediction for the minority class samples, which are often the samples for which an accurate prediction is more important. Moreover, the inclusion of a smaller number of variables in the classifiers seems a desirable property in the biomedical applications where the aim is to develop prognostic or predictive models. Many researchers argued that it is advantageous to use microarray-based classifiers that include a small number of genes (see for example [24] and references therein). The reason is that classifiers that include numerous genes can be more difficult to transfer to the clinical practice because their interpretation and practical implementation is more difficult. At the same it was shown that classifiers that include few genes can perform well in practice [24-26].

The current implementation of the NSC classifiers does not allow for case weighting so we performed random over-sampling in the attempt to give equal weight to the classes. We observed that random over-sampling had no effect on PAM, while it increased the class-imbalance bias of GM-PAM, substantially increasing the number of active variables. The reason for poor performance of GM-PAM is that the g-means used to determine the optimal threshold is, because of over-sampling, not fully cross-validated estimate as the same minority class samples are used when training and evaluating the classifier. The not properly cross-validated g-means is maximized by classifiers that use large number of variables. Although we did not perform the experiments with over-sampling for ALP and AHP, we expect that the same conclusion would still apply, as the determination of the optimal threshold would likely suffer from the same problem. In general special care is needed when the tuning parameters are determined after the training set is over-sampled; for example, Random Forests and Support Vector Machines require the optimization of the tuning parameters, which is normally done with cross-validation.

We chose to select the optimal shrinkage by maximizing the g-means of the classifiers, which is more appropriate than overall error for the assessment of the effectiveness of the classifiers trained on imbalanced data [15]. Other assessment measures were proposed for class-imbalanced data: two popular alternatives in the two-class problems are the F-measure and the area under the ROC curve (AUC), however their generalization to multi-class problems is not as straightforward as for g-means. The F-measure is a function of predictive accuracy and predictive value of the positive class, the weight given to each measure depends on a parameter that is chosen by the user. Being a function of the predictive values it is sensitive to data distributions, which is not a desirable property when data are class-imbalanced. AUC depends on the class-specific predictive accuracies, similarly to g-means. A possible advantage of g-means over AUC is its behavior when evaluating uninformative classifiers ($P(C(x^{*})=1|y=1)=P(C(x^{*})=1|y=2)$ for two classes). In this case g-means favors the classifiers that assign the same number of samples to each class, while AUC is approximately the same for all uninformative classifiers; as a consequence the estimation of the threshold parameter using AUC is very unstable when the differences between the classes are small. We considered also the maximization of the sum of the class-specific predictive accuracies ($\overset{K}{\underset{k=1}{\sum }}{\text{PA}}_{k}$), however this measure has a similar drawback as AUC as it can not distinguish between the uninformative classifiers. Experimental results for PAM showed that under the null hypothesis (and when the difference between the classes was small) this approach performed slightly worse than using g-means to determine the optimal threshold, while the results were very similar when the difference between the classes was large (data not shown).

Tibshirani *et al.*[5] proposed the procedure for adaptive choice of threshold for PAM that enables different shrinkage for each class and showed that this approach can lead to smaller number of active variables. However, Wang and Zhu [12] observed that using the adaptive threshold procedure does not change the predictive accuracy of PAM. We obtained similar results and observed that while in the multi-class classification problems sometimes the number of active variables decreases, the predictive accuracy of PAM and GM-PAM is not affected (data not shown). Therefore, the adaptive choice of threshold does not seem beneficial in decreasing the class-imbalance problem of the NSC classifiers.

In this paper we focused on PAM, ALP and AHP; others proposed further modifications to NSC methods [17,27], which were not evaluated in this study. However, we believe that all the classifiers that base their tuning on the minimization of overall error should present the same type of problems, and would benefit from using a tuning strategy based on g-means or other cost functions that are less sensitive to the class-imbalance problem.

Huang *et al.*[14] observed that the estimators of the discriminant scores for discriminant analysis are biased and derived a bias-corrected discriminant score for DLDA and DQDA. Their findings are interesting in the context of class-imbalanced high-dimensional classification, as they show that the bias of the discriminant scores depends on the class-imbalance in the training set and on the number of variables; the bias is larger in the minority class and when more variables are considered. The bias-correction outperforms the original approach, especially when the class-imbalance is large; unfortunately this approach can not be extended straightforwardly to NSC classifiers as the distribution of the estimator of the shrunken class centroid is not known.

A computational issue is the estimation of the optimal threshold. We used an approach similar to what was used for PAM [5], evaluating a fixed number of threshold values and using cross-validation. In most situations we evaluated 30 threshold values, equally spaced between 0 (all active variables) and the minimum threshold value that shrunk all the class centroids to the overall centroid (no active variables). This choice was a compromise between accuracy of estimation and computational burden, which was particularly high for AHP. However, we observed that this strategy might not be optimal in all situations since, especially for AHP, the relationship between the threshold and the number of active variables was highly nonlinear. Often the smallest positive threshold produced few active variables: the estimated number of active variables could be either very small or equal to the total number of variables, while intermediate solutions were not evaluated. This could explain why in some applications GM was not successful in improving the performance of AHP. Our observations would suggest that equally spacing the threshold values could be an effective choice when the number of variables distinguishing the classes is relatively small, as it is often the case for microarray data. In problems where many variables distinguish the classes a solution would be to equally space threshold values on the logarithmic scale, which would include more thresholds associated with a large number of active genes.

We presented the GM-NCS approach limited to the case where each class is given equal importance, but the method can be extended and incorporate different misclassification costs for each class by weighting the class-specific predictive accuracies. This approach would be useful for problems where the cost of misclassification is not equal for each class.

## Conclusion

We showed that three nearest shrunken centroid classifiers (PAM, ALP and AHP) achieve poor accuracy for the minority class when data are class-imbalanced and high-dimensional, unless the difference between classes is large. We proposed GM-NSC, a straightforward yet effective approach to diminish the class-imbalance problem of NSC classifiers, which consists in estimating the optimal amount of shrinkage by maximizing the g-means of the classifiers, rather than its overall accuracy. We used simulated and real data to show that when the NCS classifiers are biased towards the majority class the GM-NSC approach outperforms NSC, and it performs similarly to NSC otherwise. GM-NSC classifiers generally select less variables which seems a desirable property in the biomedical applications where the aim is to develop prognostic or predictive models.

Our experiments with random over-sampling showed no improvement for PAM while the class-imbalance bias of GM-PAM was increased. We therefore recommend that this strategy is not used with the NSC or GM-NSC classifiers.

## Abbreviations

NSC: nearest shrunken centroid; PAM: prediction for microarrays; DLDA: diagonal linear discriminant analysis; ALP-NSC: adaptive L∞ norm penalized NSC; AHP-NSC: adaptive hierarchically penalized NSC; PA: predictive accuracy; AUC: area under the ROC curve.

## Competing interests

Both authors declare that they have no competing interests.

## Authors’ contributions

RB derived the GM approach, performed the computations and wrote the manuscript; LL designed research and wrote the manuscript. Both authors read and approved the final manuscript.

## Supplementary Material

Additional file 1 **Derivation of the expressions for *λ***_**max**_**.** In the additional information we derive the expressions for *λ*_max_ for the classifiers considered in the paper. See text for more details. Click here for file

Additional file 2 **Classification error and the probability of classification in class 1.** In the additional file we derive the classification error as a function of a probability of classification in class *k* and class-prior probabilities. We also derive the probability of classification in class 1 for the example presented in the main text additionally assuming that the pooled variances are known. See text for more details. Click here for file

Additional file 3 **Classification results under the null hypothesis for a large number of variables (*p*=10000**) and the correlated scenario (***ρ=0.8*).** In the additional file we show predictive accuracy for class 1 (PA_1_) and PA for class 2 (PA_2_) for different levels of class-imbalance (*k*_1_) in the training set containing 100 samples. There was no difference between the classes (*μ*_2_=0). Click here for file

Additional file 4 **Classification results for a large number of variables.** In the additional file we show optimal threshold parameter (*λ*^∗^), number of active irrelevant variables (# non-info), PA for class 1 (PA_1_) and PA for class 2 (PA_2_), g-means and AUC for different levels of class-imbalance (*k*_1_) in the training set containing 100 samples for a situation where there was no difference between the classes (*μ*_2_=0; Table 1), where the difference between the classes was small (*μ*_2_=0.5; 100 variables were differentially expressed; Table 2) and where the difference between the classes was moderate (*μ*_2_=1; 100 variables were differentially expressed; Table 3). Click here for file

Additional file 5 **Classification results under the null hypothesis for a small number of variables.** The additional file shows PA for class 1 (PA_1_) and PA for class 2 (PA_2_) for different levels of class-imbalance (*k*_1_) in the training set containing 100 samples. There was no difference between the classes (*μ*_2_=0). Click here for file

Additional file 6 **Classification results under the alternative hypothesis for a small number of variables.** The additional file shows PA for class 1 (PA_1_) and PA for class 2 (PA_2_) for different levels of class-imbalance (*k*_1_) in the training set containing 100 samples. The difference between the classes was moderate (*μ*_2_=1) and 20 variables were differentially expressed (100 for AHP). Click here for file

Additional file 7 **Classification results for a small number of variables.** In the additional file we show the optimal threshold parameter (*λ*^∗^), number of active irrelevant variables (# non-info), PA for class 1 (PA_1_) and PA for class 2 (PA_2_), g-means and AUC for different levels of class-imbalance (*k*_1_) in the training set containing 100 samples. There was no difference between the classes (*μ*_2_=0; Table 1) and the differences between the classes were moderate (*μ*_2_=1; 20 variables were differentially expressed (100 for AHP); Table 2). Click here for file

Additional file 8 **Classification results for the over-sampled training set for PAM and GM-PAM.** In the additional file we show the simulation results obtained by training PAM and GM-PAM on over-sampled training sets obtained by replicating randomly selected samples from the minority class in order to obtain the class-balanced training set. Simulation settings were the same as presented in the Additional file 4. See text for more details. Click here for file

Additional file 9 **Classification results for the three class scenario.** In the additional file we show the same information as in Additional file 4 for the three class scenario. In Tables 1 and 2 we report results for the the class-balanced scenario (*n*_1_=*n*_2_=*n*_3_=100) and a large number of variables (*p*=5000) under the null and the alternative hypothesis, respectively. Table 3 reports the results for the class-imbalanced scenario (*n*_1_=*n*_3_=100, *n*_2_=20) under the null hypothesis and Table 4 reports the result under the alternative hypothesis. Results for smaller number of variables are in Table 5. Click here for file

Additional file 10 **Error rate, class specific predictive accuracies and g-means as a function of the threshold parameter for the Ivshina’s data set and prediction of ER.** In the additional file we report the error rate (left panel), accuracy for ER- class (*PA*_ER-_), accuracy for ER+ class (*PA*_ER+_) and g-means (right panel) for different values of the threshold parameter (*λ*) obtained on the Ivshina’s data set. See text for more details. Click here for file

Additional file 11 **Classification results for the gene expression data sets.** Results on the Sotiriou (2003) data set for classification of ER and Grade of the tumor (Table 1) and on the Ivshina data set for the multi class classification task (Table 2). Click here for file
