# Supplementary material for: Improved shrunken centroid classifiers for high-dimensional class-imbalanced data
Source: BMC Bioinformatics. 2013 Feb 23;14:64. doi: 10.1186/1471-2105-14-64 (PMC3687811; doi:10.1186/1471-2105-14-64)
Supplement: Additional file 1 — Derivation of the expressions for λmax. In the additional information we derive the expressions for λmax for the classifiers considered in the paper. See text for more details. [file 1471-2105-14-64-S1.pdf]

Derivation of the expressions for $\lambda_{\max}$

The shrunken centroid for all shrunken centroid based classifiers considered in the paper is defined as

$$\overline{x}'_{kj} = \overline{x}_j + \widehat{d}_{kj} \cdot m_k \cdot (s_j + s_0). \tag{1}$$

The class-centroid for variable j for class k will be equal to the overall centroid when $\widehat{d}_{kj} = 0$.

PAM

In PAM $\widehat{d}_{kj}$ is defined as

$$\widehat{d}_{kj} = \text{sgn}(d_{kj})(|d_{kj}| - \lambda)_+, \tag{2}$$

so we need to find a solution to

$$\text{sgn}(d_{kj})(|d_{kj}| - \lambda)_+ = 0, \tag{3}$$

which is $\lambda = |d_{kj}|$. In order to effectively remove variable j from the calculation of the discriminant score, we must set the threshold parameter to $\lambda = |d_{kj}|$, for each k. Setting λ to $\max_k(|d_{kj}|)$ will therefore remove the variable j from the calculation of the discriminant score. It is then obvious that when $\lambda = \max_{k,j}(|d_{kj}|)$, all class-centroids will be shrunken to the overall centroid for each variable.

ALP-NSC

According to Theorem I in Wang and Zhu (2007) the numerical solutions to p minimization problems

$$\min_{\widetilde{d}_{kj}} \frac{1}{2} \sum_{i=1}^{n} \sum_{k=1}^{K} \frac{z_{ik}}{n_k} (\widetilde{x}_{ij} - \widetilde{d}_{kj})^2 + \lambda \cdot w_j \cdot \max_k(|\widetilde{d}_{1j}|, ..., |\widetilde{d}_{Kj}|), \tag{4}$$

$j = 1, ..., p$, if there exists an indices set $C = \{k_1, ...k_r\}$, such that

$$|\widehat{d}_{k_1 j}| = ... = |\widehat{d}_{k_r j}| > |\widehat{d}_{kj}|, \text{ for } k \notin C \tag{5}$$

are

$$\widehat{d}_{kj} = \begin{cases} d_{kj} & k \notin C \\ \text{sgn}(d_{kj}) \left(\frac{1}{r} \sum_{s=1}^{r} |d_{k_s j}| - \frac{\lambda w_j}{r} \right)_+ & k \in C \end{cases}. \tag{6}$$

In order to put all class centroids to the overall centroid for variable j then $C = \{1, ..., K\}$ and the eq. 6 simplifies to

$$\widehat{d}_{kj} = \text{sgn}(d_{kj}) \left(\frac{1}{K} \sum_{k=1}^{K} |d_{kj}| - \frac{\lambda w_j}{K} \right)_+. \tag{7}$$

λ that puts $\widehat{d}_{kj}$ to zero for all k is then a solution to

$$\operatorname{sgn}(d_{kj})\left(\frac{1}{K}\sum_{k=1}^{K}|d_{kj}| - \frac{\lambda w_j}{K}\right)_+ = 0, \tag{8}$$

which yields

$$\lambda = \frac{1}{w_j}\sum_{k=1}^{K}|d_{kj}|. \tag{9}$$

In order to put all class centroids to the overall centroid for each $j = 1, ..., p$, then

$$\lambda\text{max} = \max_j\left(\frac{1}{w_j}\sum_{k=1}^{K}|d_{kj}|\right). \tag{10}$$

AHP-NSC

In Wang and Zhu (2007) the authors propose an iterative approach to estimate γ_j and θ_{kj} by using

$$\widehat{\gamma}_j = I_{(\exists k,\theta_k \neq 0)} \cdot \left(\sum_{k=1}^{K}\frac{\theta_{kj}}{\sum_{k=1}^{K}\theta_{kj}^2}d_{kj} - \frac{\lambda_\gamma w_j^\gamma}{\sum_{k=1}^{K}\theta_{kj}^2}\right)_+ \quad \text{and} \tag{11}$$

$$\widehat{\theta}_{kj} = I_{\gamma_j>0}\ \operatorname{sgn}(d_{kj})\left(\frac{|d_{kj}|}{\gamma_j} - \frac{\lambda_\theta w_{kj}^\theta}{\gamma_j^2}\right)_+, \tag{12}$$

and therefore it is necessary only to tune one of the shrinkage parameters, say λ_θ, and setting λ_γ to zero. In this case $\widehat{\gamma}_j^1$ at the first step is

$$\widehat{\gamma}_j^1 = I_{(\exists k,\theta_k \neq 0)} \cdot \left(\sum_{k=1}^{K}\frac{\theta_{kj}}{\sum_{k=1}^{K}\theta_{kj}^2}d_{kj}\right), \tag{13}$$

which after setting $\theta_{kj}^1 = \operatorname{sgn}(d_{kj})$ as proposed by the authors, yields to

$$\widehat{\gamma}_j^1 = \sum_{k=1}^{K}\frac{|d_{kj}|}{K}. \tag{14}$$

and the λ_θ that sets $\widehat{\theta}_{kj}^1$ to zero (and in the second step then also $\widehat{\gamma}_j^2$ to zero) is then a solution to

$$\operatorname{sgn}(d_{kj})\left(\frac{|d_{kj}|}{\widehat{\gamma}_j^1} - \frac{\lambda_\theta w_{kj}^\theta}{\widehat{\gamma}_j^{1^2}}\right)_+ = 0. \tag{15}$$

It is then straightforward to show that the solution to the above expression is

$$\lambda_\theta = \frac{1}{w_{kj}^\theta}\frac{1}{K}\sum_{k=1}^{K}d_{jk}^2. \tag{16}$$

This will then set the centroid for class k and variable j to the overall centroid for variable j. Setting

$$\lambda_\theta = \max_{k,j}\left(\frac{1}{w_{kj}^\theta}\frac{1}{K}\sum_{k=1}^{K}d_{jk}^2\right), \tag{17}$$

will set all class centroids to the overall centroid for all variables.
