# Supplementary material for: Improved shrunken centroid classifiers for high-dimensional class-imbalanced data
Source: BMC Bioinformatics. 2013 Feb 23;14:64. doi: 10.1186/1471-2105-14-64 (PMC3687811; doi:10.1186/1471-2105-14-64)
Supplement: Additional file 2 — Classification error and the probability of classification in class 1. In the additional file we derive the classification error as a function of a probability of classification in class k and class-prior probabilities. We also derive the probability of classification in class 1 for the example presented in the main text additionally assuming that the pooled variances are known. See text for more details. [file 1471-2105-14-64-S2.pdf]

Classification error and the probability of classification for the example presented in the main text

Let $\mathbf{x}_i = \{x_{i1}, ..., x_{ij}, ..., x_{ip}\}$ denote an observation while $\mathbf{X}_i = \{X_{i1}, ..., X_{ij}, ..., X_{ip}\}$ is used for random vector. For this special example we have $\overline{X}_{1j}$ and $\overline{X}_{2j}$ are i.i.d $N(0, \frac{1}{n_1})$ and $N(0, \frac{1}{n_2})$, respectively, and Y^* is independent of $\mathbf{X}^*$. Classification error is then

$$\begin{aligned} error &= P(\mathcal{C}(\mathbf{X}^*) \neq Y^*) \\ &= 1 - (P(\mathcal{C}(\mathbf{X}^*) = 1)P(Y^* = 1) + P(\mathcal{C}(\mathbf{X}^*) = 2)P(Y^* = 2)) \\ &= 1 - (P(\mathcal{C}(\mathbf{X}^*) = 1)\pi_1 + (1 - P(\mathcal{C}(\mathbf{X}^*) = 1))(1 - \pi_1)) \\ &= \pi_1 + P(\mathcal{C}(\mathbf{X}^*) = 1) - 2\pi_1 P(\mathcal{C}(\mathbf{X}^*) = 1), \end{aligned} \tag{1}$$

or by analogy

$$error = \pi_2 + P(\mathcal{C}(\mathbf{X}^*) = 2) - 2\pi_2 P(\mathcal{C}(\mathbf{X}^*) = 2), \tag{2}$$

where $P(\mathcal{C}(\mathbf{X}^*) = 1)$ and $P(\mathcal{C}(\mathbf{X}^*) = 2)$ are the probabilities that the classifier $\mathcal{C}$ will classify $\mathbf{X}^*$ to class 1 and class 2, respectively. Since we have that the class-variances are known and equal to one in both classes than the pooled variances are also known and equal to one, and the discriminant score for class k omitting the class prior correction is then simply

$$L_k = \sum_{j=1}^{p} (X_j^* - \overline{X}_{kj})^2. \tag{3}$$

The probability that the sample is classified in class 1, neglecting the class-prior correction is

$$P(\mathcal{C}(\mathbf{X}^*) = 1) = P(L_1 < L_2) = P\left(\sum_{j=1}^{p} (X_j^* - \overline{X}_{1j})^2 < \sum_{j=1}^{p} (X_j^* - \overline{X}_{2j})^2\right). \tag{4}$$

Denote $X_j = X_j^* - \overline{X}_{1j}$ and $Z_j = X_j^* - \overline{X}_{2j}$ and note that $X_j \sim N(0, 1 + \frac{1}{n_1})$ and $Z_j \sim N(0, 1 + \frac{1}{n_2})$. Variables $X = \sum_{j=1}^{p} X_j^2$ and $Z = \sum_{j=1}^{p} Z_j^2$ are correlated and their covariance is

$$\begin{aligned} cov(X, Z) &= cov\left(\sum_{i=1}^{p} (X_i)^2, \sum_{j=1}^{p} (Z_j)^2\right) = \sum_{i=1}^{p}\sum_{j=1}^{p} cov\left(X_i^2, Z_j^2\right) = \sum_{j=1}^{p} cov(X_j^2, Z_j^2) \\ &= \sum_{j=1}^{p} \left(var(X_j^{*2}) - 2cov(X_j^{*2}, X_j^*\overline{X}_{2j}) - 2cov(X_j^{*2}, X_j^*\overline{X}_{1j}) + 4cov(X_j^*\overline{X}_{1j}, X_j^*\overline{X}_{2j})\right) \\ &= \sum_{j=1}^{p} var(X_j^{*2}) = 2\sum_{j=1}^{p} \sigma_j^4 = 2p \end{aligned} \tag{5}$$

The variance of X is

$$\begin{aligned} var(X) &= var(\sum_{j=1}^{p} X_j^2) = \sum_{j=1}^{p} var(X_j^2) \\ &= \sum_{j=1}^{p} \left(E(X_j^2) - E(X_j^2)^2\right) = 2\sum_{j=1}^{p} (1 + \frac{1}{n_1})^2 = 2p(1 + \frac{1}{n_1})^2, \end{aligned} \tag{6}$$

and the variance of Z is then by analogy, $var(Z) = 2p(1+\frac{1}{n_2})^2$. The correlation between X and Z is then

$$\rho = \frac{cov(X,Z)}{\sqrt{var(X)var(Z)}} = \frac{2p}{2p(1+\frac{1}{n_1})(1+\frac{1}{n_2})} = \frac{1}{(1+\frac{1}{n_1})(1+\frac{1}{n_2})}. \tag{7}$$

For $p = 1$ we have

$$\begin{aligned} P(\mathcal{C}(\mathbf{X}^*) = 1) &= P\left((X^* - \overline{X}_1)^2 < (X^* - \overline{X}_2)^2\right) \\ &= P\left(|X^* - \overline{X}_1| < |X^* - \overline{X}_2|\right) \\ &= \frac{c}{2\pi}\int\int_{|X|<|Z|} e^{-\frac{c^2}{2}(x^2\frac{1+n_2}{n_2}+z^2\frac{1+n_1}{n_1}-2xz)}dzdx, \end{aligned} \tag{8}$$

where $c = \sqrt{\frac{n_1 n_2}{1+n_1+n_2}}$. The above integral can be solved analytically which yields

$$\begin{aligned} P(\mathcal{C}(\mathbf{X}^*) = 1) &= 1 - \frac{1}{\pi}\left(\arctan\left(\frac{1}{n_1}\sqrt{\frac{n_1 n_2}{1+n_1+n_2}}\right) + \right. \\ &\qquad \left. + \arctan\left(\frac{1+2n_1}{n_1}\sqrt{\frac{n_1 n_2}{1+n_1+n_2}}\right)\right). \end{aligned} \tag{9}$$

For $p > 1$ we can write

$$\begin{aligned} P(\mathcal{C}(\mathbf{X}^*) = 1) &= P\left(\sum_{j=1}^{p} X_j^2 < \sum_{j=1}^{p} Z_j^2\right) \\ &= P\left((1+\frac{1}{n_1})\sum_{j=1}^{p}\frac{X_j^2}{1+\frac{1}{n_1}} < (1+\frac{1}{n_2})\sum_{j=1}^{p}\frac{Z_j^2}{1+\frac{1}{n_2}}\right), \end{aligned} \tag{10}$$

where $U_1 = \sum_{j=1}^{p}\frac{X_j^2}{1+\frac{1}{n_1}} \sim \chi_p^2$ and $U_2 = \sum_{j=1}^{p}\frac{Z_j^2}{1+\frac{1}{n_2}} \sim \chi_p^2$. Joarder (2007) derived the probability density function (p.d.f.) of two correlated chi-square variables each with p degrees of freedom,

$$f(u_1,u_2) = \frac{2^{-(p+1)}(u_1u_2)^{\frac{p-2}{2}}e^{\frac{-(u_1+u_2)}{2(1-\rho^2)}}}{\sqrt{\pi}\Gamma(\frac{p}{2})(1-\rho^2)^{\frac{p}{2}}}\sum_{k=0}^{\infty}(1+(-1)^k)\left(\frac{\rho\sqrt{u_1u_2}}{1-\rho^2}\right)^k\frac{\Gamma\left(\frac{k+1}{2}\right)}{k!\Gamma\left(\frac{k+p}{2}\right)} \tag{11}$$

and we have,

$$P(\mathcal{C}(\mathbf{X}^*) = 1) = \int\int_{U_1<\frac{1+\frac{1}{n_2}}{1+\frac{1}{n_1}}U_2} f(u_1,u_2)du_1du_2. \tag{12}$$

The above integral can not be solved analytically but we can use numerical methods to solve it for our special case.

For a large p we can write

$$
\begin{aligned}
P(\mathcal{C}(\mathbf{X}^*)=1) &= P\left((1+\frac{1}{n_1})U_1 < (1+\frac{1}{n_2})U_2\right) \\
&= P\left(\sqrt{(1+\frac{1}{n_1})}\sqrt{2U_1} < \sqrt{(1+\frac{1}{n_2})}\sqrt{2U_2}\right), \qquad (13)
\end{aligned}
$$

where $\widetilde{X} = \sqrt{2U_1} \sim N(\sqrt{2p-1}, 1)$ and $\widetilde{Z} = \sqrt{2U_2} \sim N(\sqrt{2p-1}, 1)$, for a large p. The probability of classification in class 1 is then

$$
P(\mathcal{C}(\mathbf{X}^*)=1) = \int\int_{\widetilde{X} < \sqrt{\frac{1+\frac{1}{n_2}}{1+\frac{1}{n_1}}}\widetilde{Z}} f(\widetilde{x}, \widetilde{z}) d\widetilde{x} d\widetilde{z}, \qquad (14)
$$

where $f(\widetilde{x}, \widetilde{z})$ is a p.d.f of two correlated normally distributed random variables $\widetilde{X}$ and $\widetilde{Z}$. We can use numerical integration to solve the above integral for our special example.

Note that $cor(\sqrt{2U_1}, \sqrt{2U_2}) = cor(U_1, U_2) = cor(X, Z)$, since the correlation coefficient is invariant under monotonic increasing transformation.
