# Supplementary figures and images for: Improved shrunken centroid classifiers for high-dimensional class-imbalanced data
Source: BMC Bioinformatics. 2013 Feb 23;14:64. doi: 10.1186/1471-2105-14-64 (PMC3687811; doi:10.1186/1471-2105-14-64)

**PAM**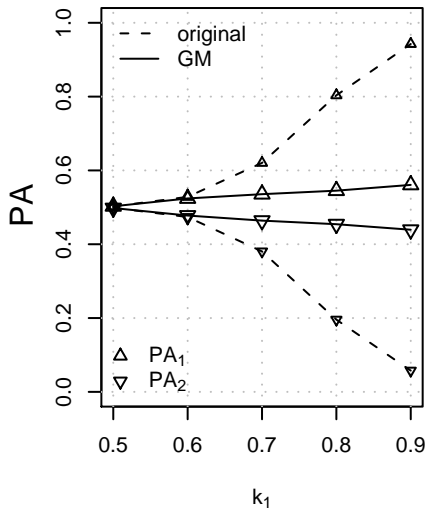**ALP**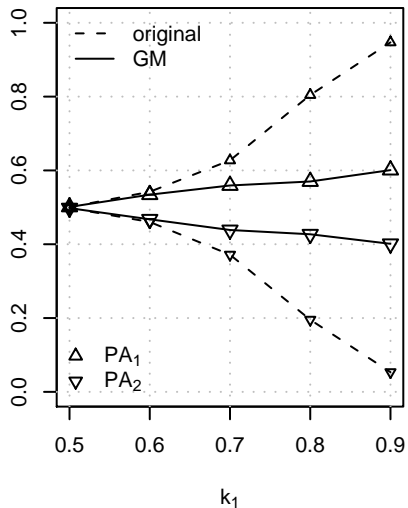**AHP**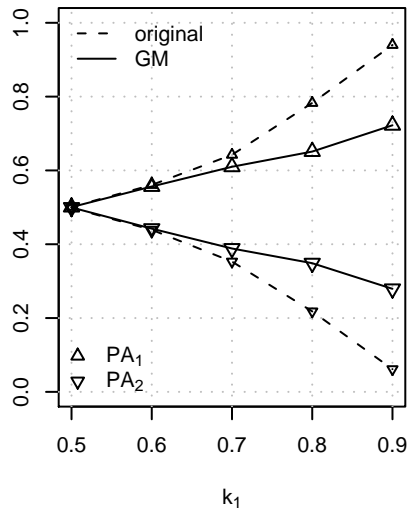

Supplement: Additional file 3 — Classification results under the null hypothesis for a large number of variables (p=10000) and the correlated scenario (ρ=0.8). In the additional file we show predictive accuracy for class 1 (PA1) and PA for class 2 (PA2) for different levels of class-imbalance (k1) in the training set containing 100 samples. There was no difference between the classes (μ2=0). [file 1471-2105-14-64-S3.pdf]

**PAM**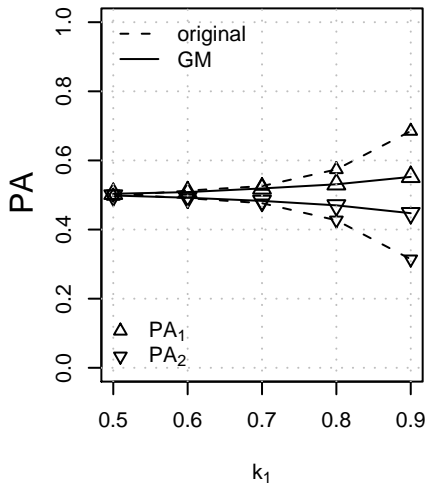**ALP**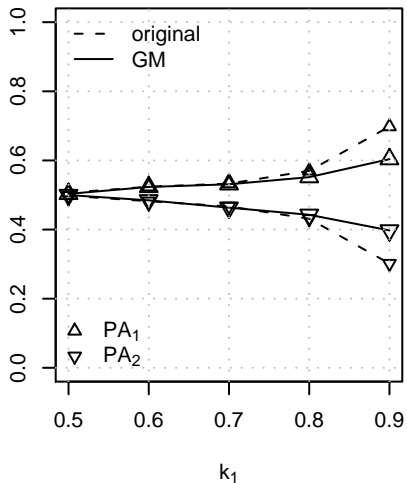**AHP**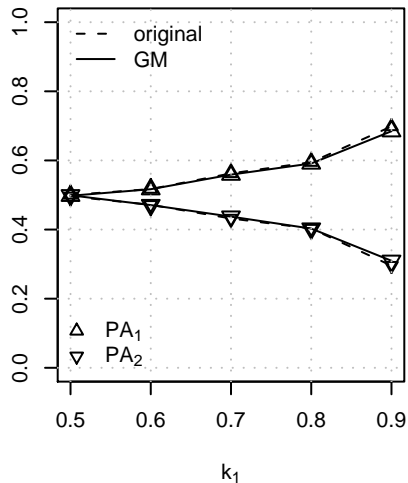

Supplement: Additional file 5 — Classification results under the null hypothesis for a small number of variables. The additional file shows PA for class 1 (PA1) and PA for class 2 (PA2) for different levels of class-imbalance (k1) in the training set containing 100 samples. There was no difference between the classes (μ2=0). [file 1471-2105-14-64-S5.pdf]

**PAM**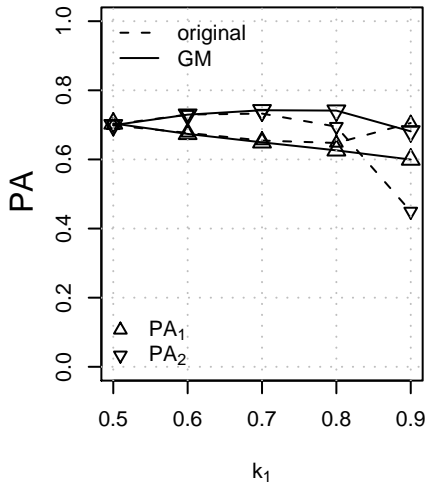**ALP**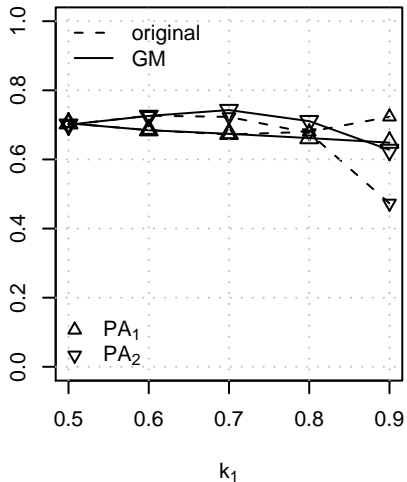**AHP**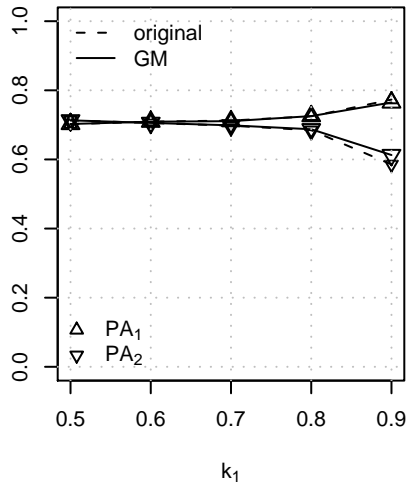

Supplement: Additional file 6 — Classification results under the alternative hypothesis for a small number of variables. The additional file shows PA for class 1 (PA1) and PA for class 2 (PA2) for different levels of class-imbalance (k1) in the training set containing 100 samples. The difference between the classes was moderate (μ2=1) and 20 variables were differentially expressed (100 for AHP). [file 1471-2105-14-64-S6.pdf]

Error

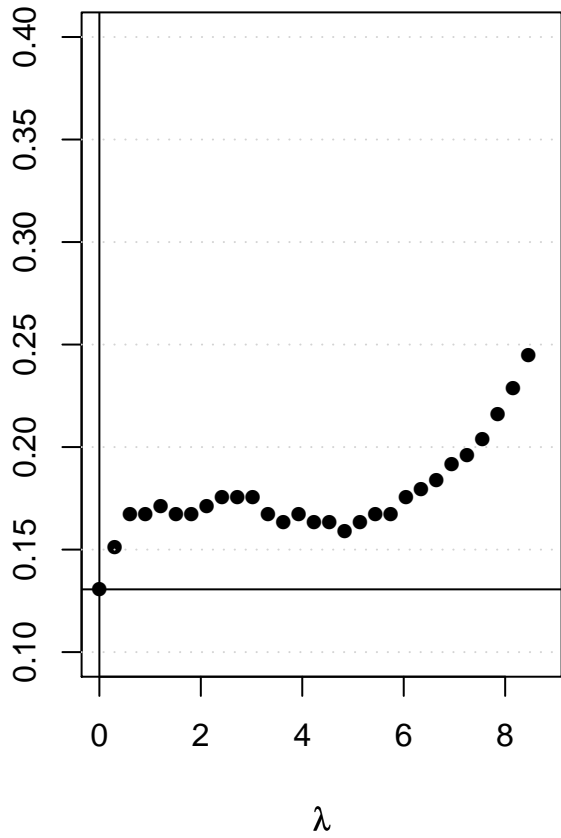

Accuracy

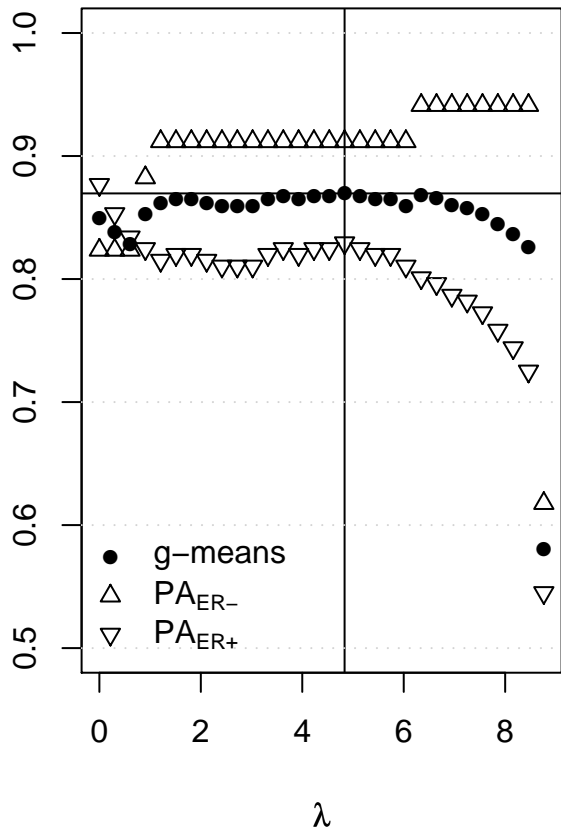

Supplement: Additional file 10 — Error rate, class specific predictive accuracies and g-means as a function of the threshold parameter for the Ivshina’s data set and prediction of ER. In the additional file we report the error rate (left panel), accuracy for ER- class (PAER-), accuracy for ER+ class (PAER+) and g-means (right panel) for different values of the threshold parameter (λ) obtained on the Ivshina’s data set. See text for more details. [file 1471-2105-14-64-S10.pdf]
