# Supplementary material for: Improved shrunken centroid classifiers for high-dimensional class-imbalanced data
Source: BMC Bioinformatics. 2013 Feb 23;14:64. doi: 10.1186/1471-2105-14-64 (PMC3687811; doi:10.1186/1471-2105-14-64)
Supplement: Additional file 4 — Classification results for a large number of variables. In the additional file we show optimal threshold parameter (λ∗), number of active irrelevant variables (# non-info), PA for class 1 (PA1) and PA for class 2 (PA2), g-means and AUC for different levels of class-imbalance (k1) in the training set containing 100 samples for a situation where there was no difference between the classes (μ2=0; Table 1), where the difference between the classes was small (μ2=0.5; 100 variables were differentially expressed; Table 2) and where the difference between the classes was moderate (μ2=1; 100 variables were differentially expressed; Table 3). [file 1471-2105-14-64-S4.pdf]

Table 1: **Classification results under the null hypothesis for a large number of variables ($p = 10000$) and the correlated scenario ($\rho = 0.8$).** The table shows optimal threshold parameter (λ^*), number of active irrelevant variables (# non-info), PA for class 1 (PA_1) and PA for class 2 (PA_2), g-means and AUC for different levels of class-imbalance (k_1) in the training set containing 100 samples. There was no difference between the classes ($\mu_2 = 0$).

k_1	Method	λ^*	# non-info	PA_1	PA_2	g-means	AUC
0.5	PAM	1.25	1714.84	0.5	0.5	0.5	0.5
		(0.66)	(3024.51)	(0.04)	(0.04)	(0.02)	(0.02)
	GM-PAM	1.21	1894.69	0.5	0.5	0.5	0.5
		(0.67)	(3175.92)	(0.04)	(0.04)	(0.02)	(0.02)
	ALP	3.19	2182.79	0.5	0.5	0.5	0.5
		(2.46)	(3511.75)	(0.05)	(0.05)	(0.02)	(0.02)
	GM-ALP	3.06	2263.77	0.5	0.5	0.5	0.5
		(2.43)	(3544.79)	(0.05)	(0.05)	(0.02)	(0.02)
	AHP	2.73	1833.34	0.5	0.5	0.5	0.5
		(2.3)	(3455.85)	(0.04)	(0.04)	(0.02)	(0.02)
	GM-AHP	2.53	1958.54	0.5	0.5	0.5	0.5
		(2.27)	(3518.79)	(0.04)	(0.04)	(0.02)	(0.02)
0.6	PAM	1.11	2317.71	0.53	0.47	0.5	0.5
		(0.69)	(3512.03)	(0.05)	(0.06)	(0.02)	(0.01)
	GM-PAM	1.18	1820.87	0.52	0.48	0.5	0.5
		(0.64)	(3026.06)	(0.05)	(0.05)	(0.02)	(0.01)
	ALP	2.75	2770.38	0.54	0.46	0.5	0.5
		(2.54)	(3779.34)	(0.06)	(0.06)	(0.02)	(0.02)
	GM-ALP	3.06	1987.98	0.53	0.47	0.5	0.5
		(2.42)	(3190.02)	(0.05)	(0.05)	(0.02)	(0.02)
	AHP	2.58	2129.23	0.56	0.44	0.49	0.5
		(2.39)	(3685.05)	(0.05)	(0.05)	(0.02)	(0.02)
	GM-AHP	2.73	1770.8	0.56	0.44	0.49	0.5
		(2.34)	(3423.47)	(0.05)	(0.05)	(0.02)	(0.02)
0.7	PAM	0.59	5481.04	0.62	0.38	0.48	0.5
		(0.71)	(4198.91)	(0.09)	(0.09)	(0.03)	(0.02)
	GM-PAM	1.25	1225.24	0.54	0.46	0.49	0.5
		(0.57)	(2230.39)	(0.06)	(0.06)	(0.02)	(0.02)
	ALP	1.49	5080.11	0.63	0.37	0.47	0.5
		(2.18)	(4264.38)	(0.09)	(0.09)	(0.03)	(0.02)
	GM-ALP	3.21	1584.57	0.56	0.44	0.49	0.5
		(2.34)	(2782.6)	(0.08)	(0.08)	(0.02)	(0.02)
	AHP	1.8	2930.47	0.64	0.35	0.47	0.5
		(2.07)	(4052.71)	(0.07)	(0.07)	(0.03)	(0.02)
	GM-AHP	3.01	1226.88	0.61	0.39	0.48	0.5
		(2.19)	(2838.52)	(0.07)	(0.07)	(0.02)	(0.02)
0.8	PAM	0.06	9230.38	0.8	0.2	0.39	0.5
		(0.21)	(1655.79)	(0.06)	(0.06)	(0.04)	(0.02)
	GM-PAM	1.37	762.37	0.55	0.45	0.49	0.5
		(0.52)	(1626.6)	(0.08)	(0.08)	(0.02)	(0.02)
	ALP	0.16	8866.96	0.8	0.2	0.39	0.5
		(0.66)	(2627.17)	(0.06)	(0.06)	(0.04)	(0.02)
	GM-ALP	3.78	776.08	0.57	0.43	0.48	0.5
		(2.15)	(1792.69)	(0.09)	(0.09)	(0.03)	(0.02)
	AHP	0.74	5453.21	0.78	0.22	0.41	0.5
		(1.61)	(4413.4)	(0.07)	(0.07)	(0.04)	(0.02)
	GM-AHP	3.71	477.33	0.65	0.35	0.47	0.5
		(2.13)	(1703.37)	(0.08)	(0.08)	(0.03)	(0.02)
0.9	PAM	0.02	9616.19	0.94	0.06	0.23	0.5
		(0.05)	(725.82)	(0.02)	(0.02)	(0.04)	(0.02)
	GM-PAM	1.4	524.32	0.56	0.44	0.49	0.5
		(0.45)	(1163.06)	(0.1)	(0.09)	(0.03)	(0.01)
	ALP	0.02	9642.75	0.95	0.05	0.22	0.5
		(0.08)	(1350.17)	(0.02)	(0.02)	(0.04)	(0.02)
	GM-ALP	3.93	495.47	0.6	0.4	0.48	0.5
		(2.01)	(1234.67)	(0.11)	(0.11)	(0.05)	(0.02)
	AHP	0.13	7091.47	0.94	0.06	0.23	0.5
		(0.23)	(3927.21)	(0.02)	(0.03)	(0.04)	(0.02)
	GM-AHP	4.12	263.35	0.72	0.28	0.43	0.5
		(1.94)	(1180.74)	(0.11)	(0.11)	(0.07)	(0.02)

Table 2: **Classification results under the alternative hypothesis for a large number of variables ($p = 10000$) and the correlated scenario ($\rho = 0.8$).** The table shows optimal threshold parameter (λ^*), number of active relevant variables (# info), number of active irrelevant variables (# non-info), PA for class 1 (PA_1) and PA for class 2 (PA_2), g-means and AUC for different levels of class-imbalance (k_1) in the training set containing 100 samples. The differences between the classes were small ($\mu_2 = 0.5$; 100 variables were differentially expressed).

k_1	Method	λ^*	# info	# non-info	PA_1	PA_2	g-means	AUC
0.5	PAM	1.34	43.04	1438.42	0.55	0.55	0.55	0.57
		(0.7)	(43.71)	(2719.85)	(0.06)	(0.06)	(0.05)	(0.06)
	GM-PAM	1.28	46.48	1601.15	0.55	0.55	0.55	0.57
		(0.71)	(44.21)	(2861.99)	(0.06)	(0.06)	(0.05)	(0.06)
	ALP	3.73	53.07	1485.14	0.56	0.56	0.56	0.57
		(2.88)	(42.37)	(2773.9)	(0.06)	(0.06)	(0.05)	(0.05)
	GM-ALP	3.57	55.12	1640.82	0.56	0.56	0.55	0.57
		(2.84)	(42.02)	(2940.85)	(0.06)	(0.06)	(0.04)	(0.05)
	AHP	3.75	42.01	1111.68	0.55	0.55	0.55	0.57
		(3.3)	(40.93)	(2673.99)	(0.06)	(0.06)	(0.05)	(0.05)
	GM-AHP	3.58	44.02	1209.46	0.55	0.55	0.55	0.57
		(3.27)	(41.21)	(2782.07)	(0.06)	(0.06)	(0.04)	(0.05)
0.6	PAM	1.2	50.96	2043.56	0.57	0.53	0.55	0.57
		(0.74)	(44.64)	(3225.07)	(0.06)	(0.08)	(0.05)	(0.06)
	GM-PAM	1.26	47.86	1569.16	0.56	0.54	0.55	0.57
		(0.69)	(44)	(2782.5)	(0.06)	(0.08)	(0.05)	(0.06)
	ALP	3.43	57.84	2040.27	0.58	0.53	0.55	0.57
		(3.04)	(42.35)	(3362.6)	(0.06)	(0.08)	(0.05)	(0.05)
	GM-ALP	3.57	54.93	1629.5	0.57	0.53	0.55	0.57
		(2.9)	(42.79)	(2986.19)	(0.06)	(0.08)	(0.05)	(0.05)
	AHP	3.35	46.04	1407.81	0.59	0.51	0.55	0.57
		(3.3)	(41.47)	(3003.61)	(0.05)	(0.08)	(0.05)	(0.05)
	GM-AHP	3.42	44.3	1200.94	0.59	0.51	0.55	0.57
		(3.14)	(41.1)	(2782.2)	(0.05)	(0.08)	(0.05)	(0.05)
0.7	PAM	0.77	68.79	4385.98	0.63	0.45	0.52	0.56
		(0.78)	(42.59)	(4166.54)	(0.09)	(0.12)	(0.05)	(0.05)
	GM-PAM	1.33	39.13	1045.2	0.56	0.52	0.54	0.56
		(0.6)	(42.62)	(2132.2)	(0.07)	(0.09)	(0.05)	(0.06)
	ALP	1.92	73.09	4191.88	0.64	0.44	0.52	0.55
		(2.44)	(39.42)	(4228)	(0.08)	(0.12)	(0.05)	(0.05)
	GM-ALP	3.66	44.77	1188.64	0.59	0.5	0.54	0.56
		(2.61)	(43.02)	(2306.65)	(0.08)	(0.1)	(0.05)	(0.05)
	AHP	2.15	57.85	2624.07	0.66	0.42	0.52	0.55
		(2.67)	(41.75)	(3929.2)	(0.06)	(0.1)	(0.05)	(0.05)
	GM-AHP	3.62	33.71	819.84	0.63	0.46	0.53	0.56
		(2.8)	(38.32)	(2256.46)	(0.07)	(0.1)	(0.05)	(0.06)
0.8	PAM	0.07	98.28	9028.45	0.81	0.23	0.42	0.54
		(0.2)	(9.81)	(1840.1)	(0.06)	(0.07)	(0.04)	(0.03)
	GM-PAM	1.34	31.84	839.52	0.56	0.5	0.52	0.55
		(0.54)	(41.02)	(1744.37)	(0.08)	(0.1)	(0.05)	(0.06)
	ALP	0.19	95.92	8322.8	0.81	0.23	0.42	0.54
		(0.64)	(17.23)	(2953.94)	(0.06)	(0.08)	(0.05)	(0.03)
	GM-ALP	3.64	36.11	817.92	0.59	0.47	0.52	0.54
		(2.26)	(41.69)	(1757.85)	(0.09)	(0.11)	(0.05)	(0.05)
	AHP	0.85	79	4987.73	0.79	0.26	0.45	0.55
		(1.84)	(34.11)	(4442.68)	(0.07)	(0.1)	(0.06)	(0.04)
	GM-AHP	3.94	24.07	493.42	0.67	0.39	0.51	0.55
		(2.52)	(34.84)	(1727.62)	(0.08)	(0.11)	(0.06)	(0.06)
0.9	PAM	0.03	98.99	9463.69	0.94	0.07	0.25	0.53
		(0.05)	(5.59)	(809.28)	(0.02)	(0.03)	(0.05)	(0.02)
	GM-PAM	1.37	20.16	629.3	0.57	0.45	0.5	0.52
		(0.48)	(35.45)	(1339.82)	(0.1)	(0.11)	(0.05)	(0.05)
	ALP	0.02	98.98	9497.97	0.95	0.06	0.24	0.52
		(0.07)	(7.41)	(1381.33)	(0.02)	(0.02)	(0.04)	(0.02)
	GM-ALP	3.79	20.71	507.53	0.61	0.42	0.49	0.52
		(2.07)	(34.08)	(1129.18)	(0.11)	(0.12)	(0.06)	(0.04)
	AHP	0.15	83.42	6742.98	0.94	0.07	0.26	0.53
		(0.25)	(31.57)	(3998.91)	(0.03)	(0.03)	(0.05)	(0.03)
	GM-AHP	4.17	11.25	339.6	0.73	0.3	0.45	0.52
		(2.05)	(25.83)	(1418.47)	(0.11)	(0.12)	(0.08)	(0.05)

Table 3: **Classification results under the alternative hypothesis for a large number of variables ($p = 10000$) and the correlated scenario ($\rho = 0.8$).** The table shows optimal threshold parameter (λ^*), number of active relevant variables (# info), number of active irrelevant variables (# non-info), PA for class 1 (PA_1) and PA for class 2 (PA_2), g-means and AUC for different levels of class-imbalance (k_1) in the training set containing 100 samples. The differences between the classes were moderate ($\mu_2 = 1$; 100 variables were differentially expressed).

k_1	Method	λ^*	# info	# non-info	PA_1	PA_2	g-means	AUC
0.5	PAM	2.16	52.63	403.88	0.7	0.7	0.7	0.77
		(0.93)	(42.1)	(1303.07)	(0.05)	(0.05)	(0.03)	(0.04)
	GM-PAM	2.09	56.11	449.75	0.7	0.7	0.7	0.77
		(0.94)	(42.3)	(1379.52)	(0.05)	(0.05)	(0.03)	(0.04)
	ALP	10.61	60.5	223.52	0.71	0.7	0.7	0.72
		(6.74)	(34.86)	(819.07)	(0.04)	(0.04)	(0.02)	(0.03)
	GM-ALP	10.11	63.59	283.23	0.71	0.7	0.7	0.72
		(6.82)	(34.79)	(967.82)	(0.04)	(0.04)	(0.02)	(0.03)
	AHP	14.94	56.54	190.5	0.7	0.7	0.7	0.73
		(10.98)	(33.92)	(1190.91)	(0.05)	(0.05)	(0.03)	(0.03)
	GM-AHP	14.06	60.43	221.09	0.7	0.7	0.7	0.73
		(10.95)	(34)	(1273.27)	(0.05)	(0.05)	(0.03)	(0.03)
0.6	PAM	1.97	59.15	493.12	0.68	0.72	0.7	0.77
		(0.93)	(43.52)	(1380.39)	(0.04)	(0.06)	(0.03)	(0.03)
	GM-PAM	1.99	59.21	408.9	0.68	0.72	0.7	0.77
		(0.9)	(42.94)	(1160.9)	(0.04)	(0.05)	(0.03)	(0.03)
	ALP	8.28	67.95	338.95	0.69	0.71	0.7	0.72
		(6.01)	(35.86)	(1273.09)	(0.05)	(0.06)	(0.03)	(0.03)
	GM-ALP	8.16	69.29	244.88	0.69	0.71	0.7	0.72
		(5.92)	(35.21)	(812.89)	(0.04)	(0.05)	(0.03)	(0.03)
	AHP	13.59	57.42	214.72	0.71	0.7	0.7	0.74
		(10.68)	(33.67)	(1272.28)	(0.04)	(0.05)	(0.03)	(0.03)
	GM-AHP	13.32	59.31	86.69	0.71	0.7	0.7	0.74
		(10.4)	(33.07)	(653.92)	(0.04)	(0.05)	(0.03)	(0.03)
0.7	PAM	1.36	76.46	1608.89	0.67	0.68	0.67	0.74
		(0.89)	(38.92)	(2804.46)	(0.06)	(0.12)	(0.05)	(0.06)
	GM-PAM	1.76	64.3	480.41	0.65	0.73	0.69	0.76
		(0.82)	(42.71)	(1184.06)	(0.05)	(0.08)	(0.03)	(0.04)
	ALP	4.79	83.59	1364.82	0.69	0.67	0.68	0.72
		(4.69)	(30.65)	(2783.01)	(0.06)	(0.12)	(0.05)	(0.05)
	GM-ALP	6.77	73.61	322.1	0.67	0.72	0.69	0.72
		(4.91)	(35.34)	(1054.82)	(0.05)	(0.07)	(0.03)	(0.04)
	AHP	11.73	59.2	496.52	0.73	0.66	0.69	0.73
		(10.81)	(36.69)	(1936.22)	(0.04)	(0.09)	(0.05)	(0.04)
	GM-AHP	12.2	56.41	64.86	0.72	0.68	0.7	0.74
		(10.1)	(34.89)	(420.45)	(0.04)	(0.06)	(0.03)	(0.03)
0.8	PAM	0.26	98.95	7138.77	0.8	0.39	0.54	0.66
		(0.41)	(8.87)	(3491.76)	(0.08)	(0.17)	(0.09)	(0.06)
	GM-PAM	1.53	66.76	496.15	0.64	0.73	0.68	0.75
		(0.66)	(41.32)	(1068.22)	(0.07)	(0.1)	(0.05)	(0.06)
	ALP	0.82	99.66	6115.45	0.8	0.41	0.55	0.66
		(1.43)	(4.75)	(4264.64)	(0.08)	(0.19)	(0.1)	(0.06)
	GM-ALP	4.96	74.6	334.81	0.66	0.71	0.68	0.72
		(3.48)	(35.67)	(767.47)	(0.07)	(0.1)	(0.05)	(0.05)
	AHP	5.06	78.41	2910.9	0.79	0.49	0.6	0.69
		(7.98)	(33.83)	(4240.07)	(0.06)	(0.18)	(0.1)	(0.06)
	GM-AHP	8.69	52.01	94.03	0.73	0.65	0.68	0.73
		(7.08)	(34.99)	(638.55)	(0.05)	(0.1)	(0.05)	(0.05)
