# Supplementary material for: Improved shrunken centroid classifiers for high-dimensional class-imbalanced data
Source: BMC Bioinformatics. 2013 Feb 23;14:64. doi: 10.1186/1471-2105-14-64 (PMC3687811; doi:10.1186/1471-2105-14-64)
Supplement: Additional file 7 — Classification results for a small number of variables. In the additional file we show the optimal threshold parameter (λ∗), number of active irrelevant variables (# non-info), PA for class 1 (PA1) and PA for class 2 (PA2), g-means and AUC for different levels of class-imbalance (k1) in the training set containing 100 samples. There was no difference between the classes (μ2=0; Table 1) and the differences between the classes were moderate (μ2=1; 20 variables were differentially expressed (100 for AHP); Table 2). [file 1471-2105-14-64-S7.pdf]

Table 1: **Classification results under the null hypothesis for a small number of variables ($p = 1000$) and the correlated scenario ($\rho = 0.8$).** The table shows optimal threshold parameter (λ^*), number of active irrelevant variables (# non-info), PA for class 1 (PA_1) and PA for class 2 (PA_2), g-means and AUC for different levels of class-imbalance (k_1) in the training set containing 100 samples. There was no difference between the classes ($\mu_2 = 0$).

k_1	Method	λ^*	# non-info	PA_1	PA_2	g-means	AUC
0.5	PAM	0.97	208.71	0.5	0.5	0.5	0.5
		(0.52)	(7.34)	(0.04)	(0.04)	(0.02)	(0.02)
	GM-PAM	0.89	5.1	0.5	0.5	0.5	0.5
		(0.54)	(7.82)	(0.04)	(0.04)	(0.02)	(0.02)
	ALP	2.06	257.16	0.51	0.5	0.5	0.5
		(1.61)	(384.11)	(0.04)	(0.04)	(0.01)	(0.02)
	GM-ALP	1.86	5.64	0.5	0.5	0.5	0.5
		(1.52)	(8.57)	(0.04)	(0.05)	(0.01)	(0.02)
	AHP	1.62	150.2	0.5	0.5	0.5	0.5
		(1.38)	(292.24)	(0.04)	(0.05)	(0.02)	(0.02)
	GM-AHP	1.47	25.3	0.5	0.5	0.5	0.5
		(1.33)	(40.05)	(0.04)	(0.05)	(0.02)	(0.02)
0.6	PAM	0.92	246.51	0.51	0.49	0.5	0.5
		(0.54)	(7.58)	(0.04)	(0.04)	(0.02)	(0.02)
	GM-PAM	0.97	3.95	0.51	0.49	0.5	0.5
		(0.52)	(7.01)	(0.04)	(0.04)	(0.02)	(0.02)
	ALP	1.69	305.12	0.52	0.48	0.5	0.5
		(1.65)	(366.91)	(0.05)	(0.04)	(0.02)	(0.02)
	GM-ALP	1.64	6.56	0.52	0.48	0.5	0.5
		(1.64)	(8.11)	(0.05)	(0.05)	(0.02)	(0.02)
	AHP	1.54	188.94	0.52	0.47	0.49	0.49
		(1.15)	(348.92)	(0.05)	(0.04)	(0.01)	(0.02)
	GM-AHP	1.55	17.9	0.52	0.47	0.49	0.49
		(1.13)	(36.04)	(0.05)	(0.04)	(0.01)	(0.02)
0.7	PAM	0.86	286.96	0.53	0.48	0.5	0.5
		(0.57)	(8.4)	(0.05)	(0.05)	(0.02)	(0.02)
	GM-PAM	0.94	4.43	0.52	0.48	0.5	0.5
		(0.52)	(7.57)	(0.05)	(0.05)	(0.02)	(0.02)
	ALP	1.84	240.06	0.53	0.47	0.5	0.5
		(1.41)	(336.46)	(0.05)	(0.05)	(0.01)	(0.02)
	GM-ALP	1.98	3.56	0.53	0.46	0.49	0.49
		(1.46)	(6.37)	(0.05)	(0.05)	(0.02)	(0.02)
	AHP	1.32	256.34	0.56	0.43	0.49	0.5
		(1.33)	(386.09)	(0.05)	(0.05)	(0.02)	(0.02)
	GM-AHP	1.18	27.76	0.56	0.44	0.49	0.5
		(1.15)	(40.11)	(0.05)	(0.05)	(0.02)	(0.02)
0.8	PAM	0.47	572.72	0.57	0.43	0.49	0.5
		(0.55)	(8.87)	(0.07)	(0.07)	(0.02)	(0.02)
	GM-PAM	0.96	4.13	0.53	0.47	0.5	0.5
		(0.5)	(7.18)	(0.06)	(0.06)	(0.02)	(0.02)
	ALP	1.22	465.28	0.57	0.43	0.49	0.5
		(1.5)	(428.56)	(0.05)	(0.06)	(0.02)	(0.02)
	GM-ALP	1.64	5.44	0.55	0.44	0.49	0.5
		(1.56)	(8.27)	(0.05)	(0.04)	(0.02)	(0.02)
	AHP	1.31	294.78	0.6	0.4	0.49	0.5
		(1.39)	(432.51)	(0.06)	(0.05)	(0.02)	(0.02)
	GM-AHP	1.64	22.58	0.59	0.4	0.49	0.5
		(1.45)	(39.77)	(0.05)	(0.05)	(0.02)	(0.01)
0.9	PAM	0.05	926.09	0.68	0.31	0.46	0.5
		(0.16)	(4)	(0.06)	(0.05)	(0.02)	(0.02)
	GM-PAM	0.96	3.94	0.55	0.45	0.49	0.5
		(0.5)	(7.19)	(0.08)	(0.08)	(0.02)	(0.02)
	ALP	0.1	860.06	0.7	0.3	0.45	0.5
		(0.27)	(254.66)	(0.06)	(0.06)	(0.03)	(0.02)
	GM-ALP	1.88	5.04	0.6	0.4	0.48	0.5
		(1.58)	(7.58)	(0.08)	(0.09)	(0.03)	(0.02)
	AHP	1.17	252.48	0.7	0.3	0.45	0.5
		(1.44)	(362.18)	(0.05)	(0.06)	(0.03)	(0.02)
	GM-AHP	1.2	27.32	0.68	0.31	0.46	0.5
		(1.38)	(40.65)	(0.05)	(0.06)	(0.03)	(0.02)

Table 2: **Classification results under the alternative hypothesis for a small number of variables ($p = 1000$) and the correlated scenario ($\rho = 0.8$).** The table shows optimal threshold parameter (λ^*), number of active relevant variables (# info), number of active irrelevant variables (# non-info), PA for class 1 (PA_1) and PA for class 2 (PA_2), g-means and AUC for different levels of class-imbalance (k_1) in the training set containing 100 samples. The differences between the classes were moderate ($\mu_2 = 1$; 20 variables were differentially expressed (100 for AHP)).

k_1	Method	λ^*	# info	# non-info	PA_1	PA_2	g-means	AUC
0.5	PAM	2.17	12.52	21.84	0.7	0.7	0.7	0.77
		(0.82)	(7.6)	(82.87)	(0.05)	(0.05)	(0.03)	(0.04)
	GM-PAM	2.14	12.43	22.15	0.7	0.7	0.7	0.77
		(0.81)	(7.57)	(88.05)	(0.05)	(0.05)	(0.03)	(0.04)
	ALP	10.57	13.84	26.08	0.7	0.7	0.7	0.74
		(6.35)	(6)	(138.93)	(0.05)	(0.05)	(0.03)	(0.04)
	GM-ALP	10.11	14.36	27.1	0.7	0.7	0.7	0.74
		(6.41)	(5.96)	(138.91)	(0.05)	(0.05)	(0.03)	(0.04)
	AHP	13.9	55.86	58.34	0.7	0.71	0.71	0.74
		(10.91)	(35.6)	(215.27)	(0.03)	(0.04)	(0.01)	(0.02)
	GM-AHP	13.6	57.54	58.34	0.7	0.71	0.71	0.74
		(10.58)	(34.73)	(215.27)	(0.03)	(0.04)	(0.01)	(0.02)
0.6	PAM	2.05	13.08	16.42	0.68	0.73	0.7	0.78
		(0.81)	(7.55)	(52.05)	(0.05)	(0.05)	(0.03)	(0.04)
	GM-PAM	2.11	12.34	13.89	0.67	0.73	0.7	0.77
		(0.78)	(7.81)	(49.1)	(0.05)	(0.05)	(0.03)	(0.03)
	ALP	9.61	15.4	6.32	0.68	0.73	0.7	0.74
		(5.31)	(5.93)	(23.61)	(0.04)	(0.05)	(0.03)	(0.03)
	GM-ALP	9.75	15.08	6.32	0.68	0.73	0.7	0.74
		(5.27)	(6.19)	(23.61)	(0.04)	(0.05)	(0.03)	(0.03)
	AHP	13.96	60.26	81.64	0.71	0.71	0.71	0.74
		(11.22)	(33.12)	(245.27)	(0.04)	(0.04)	(0.01)	(0.02)
	GM-AHP	13.6	64.86	80.52	0.71	0.71	0.71	0.74
		(11.09)	(30.14)	(245.49)	(0.04)	(0.04)	(0.01)	(0.02)
0.7	PAM	1.64	14.99	43.47	0.65	0.73	0.69	0.76
		(0.72)	(7.42)	(134.27)	(0.05)	(0.07)	(0.04)	(0.06)
	GM-PAM	1.8	13.91	27.79	0.65	0.74	0.69	0.77
		(0.74)	(7.64)	(89.27)	(0.05)	(0.06)	(0.04)	(0.05)
	ALP	4.78	17.6	69.16	0.67	0.72	0.7	0.73
		(3.9)	(4.99)	(207.88)	(0.05)	(0.08)	(0.05)	(0.06)
	GM-ALP	5.86	16.44	14.24	0.67	0.74	0.71	0.74
		(4.19)	(5.76)	(38.51)	(0.06)	(0.06)	(0.04)	(0.05)
	AHP	12.75	47.36	67.18	0.71	0.7	0.7	0.74
		(10.1)	(33.68)	(216.58)	(0.04)	(0.04)	(0.02)	(0.02)
	GM-AHP	11.83	56.88	52.36	0.71	0.7	0.7	0.74
		(9.9)	(32.36)	(180.61)	(0.04)	(0.04)	(0.02)	(0.02)
0.8	PAM	1.12	17.36	164.61	0.65	0.69	0.67	0.74
		(0.66)	(5.89)	(304.3)	(0.05)	(0.13)	(0.07)	(0.08)
	GM-PAM	1.53	14.29	51.69	0.63	0.74	0.68	0.76
		(0.62)	(7.52)	(155.81)	(0.06)	(0.1)	(0.05)	(0.07)
	ALP	2.96	18.24	123.66	0.68	0.68	0.67	0.73
		(2.36)	(4.61)	(252.95)	(0.05)	(0.11)	(0.06)	(0.07)
	GM-ALP	4.88	15.72	58.5	0.66	0.71	0.68	0.72
		(3.33)	(5.99)	(174.25)	(0.05)	(0.1)	(0.05)	(0.05)
	AHP	8.78	43.94	87.58	0.72	0.68	0.7	0.74
		(6.84)	(34.95)	(249.36)	(0.04)	(0.06)	(0.02)	(0.02)
	GM-AHP	8.83	50.14	67.82	0.73	0.69	0.7	0.74
		(6.7)	(34.74)	(218.97)	(0.04)	(0.05)	(0.02)	(0.02)
0.9	PAM	0.26	19.83	705.08	0.71	0.45	0.55	0.61
		(0.4)	(1.26)	(374.47)	(0.06)	(0.17)	(0.08)	(0.09)
	GM-PAM	1.21	12.74	108.62	0.6	0.68	0.63	0.7
		(0.53)	(8.22)	(228.62)	(0.07)	(0.16)	(0.08)	(0.11)
	ALP	0.75	19.74	524.76	0.72	0.47	0.57	0.64
		(1.02)	(1.7)	(423.88)	(0.05)	(0.18)	(0.1)	(0.09)
	GM-ALP	2.81	14.48	154.28	0.65	0.63	0.63	0.68
		(2.19)	(7.3)	(303.08)	(0.08)	(0.18)	(0.1)	(0.1)
	AHP	3.16	62.54	238.2	0.77	0.59	0.67	0.73
		(4.16)	(39.48)	(363.93)	(0.05)	(0.09)	(0.05)	(0.03)
	GM-AHP	3.8	56.6	87.86	0.76	0.61	0.68	0.73
		(3.86)	(37.87)	(219.83)	(0.05)	(0.09)	(0.04)	(0.04)
