# Supplementary material for: Improved shrunken centroid classifiers for high-dimensional class-imbalanced data
Source: BMC Bioinformatics. 2013 Feb 23;14:64. doi: 10.1186/1471-2105-14-64 (PMC3687811; doi:10.1186/1471-2105-14-64)

Table 1: **Classification results obtained after over-sampling the training set.** The table shows optimal threshold parameter (λ^*), number of active irrelevant variables (# non-info), predictive accuracy (PA), PA for class 1 (PA_1) and PA for class 2 (PA_2), g-means and AUC for different levels of class-imbalance (k_1) in the training set containing 100 samples and difference between the classes (μ_2). The training set was over-sampled in order to obtain a class-balanced training set. There were 10,000 variables ($p = 10,000$) and the correlation coefficient for the variables within the same block containing 100 variables was set to 0.8. See text for more details.

μ_2	k_1		PA	PA_1	PA_2	g-means	AUC	# info	# non-info	λ
0	0.5	PAM	0.5	0.5	0.5	0.5	0.5	22.04	2019.31	1.18
			(0.01)	(0.04)	(0.04)	(0.01)	(0.02)	(37.49)	(3328.64)	(0.67)
		GM-PAM	0.5	0.5	0.5	0.5	0.5	24.71	2242.56	1.13
			(0.02)	(0.04)	(0.05)	(0.02)	(0.02)	(38.43)	(3436.9)	(0.68)
	0.6	PAM	0.5	0.62	0.38	0.48	0.5	51.87	4795.4	0.68
			(0.01)	(0.06)	(0.06)	(0.02)	(0.02)	(42.05)	(3573.33)	(0.7)
		GM-PAM	0.5	0.62	0.37	0.48	0.5	54.18	5037.92	0.64
			(0.01)	(0.06)	(0.06)	(0.02)	(0.02)	(41.99)	(3592.46)	(0.69)
	0.7	PAM	0.5	0.74	0.27	0.44	0.5	61.92	5799.99	0.49
			(0.01)	(0.05)	(0.06)	(0.03)	(0.02)	(38.43)	(2915.88)	(0.54)
		GM-PAM	0.5	0.74	0.27	0.44	0.5	62.65	5911.31	0.48
			(0.01)	(0.05)	(0.06)	(0.03)	(0.02)	(38.56)	(2939.22)	(0.54)
	0.8	PAM	0.5	0.84	0.15	0.36	0.5	71.32	6847.44	0.37
			(0.01)	(0.04)	(0.04)	(0.04)	(0.02)	(31.71)	(2450.68)	(0.33)
		GM-PAM	0.5	0.84	0.15	0.36	0.5	71.52	6921.11	0.36
			(0.01)	(0.04)	(0.04)	(0.04)	(0.02)	(31.75)	(2483.02)	(0.33)
	0.9	PAM	0.5	0.95	0.05	0.22	0.5	61.37	6026.48	0.69
			(0.01)	(0.02)	(0.02)	(0.04)	(0.02)	(37.62)	(2742.67)	(0.56)
		GM-PAM	0.5	0.95	0.05	0.21	0.5	62.69	6127.63	0.67
			(0.01)	(0.02)	(0.02)	(0.04)	(0.02)	(36.69)	(2651.88)	(0.53)
0.5	0.5	PAM	0.55	0.55	0.56	0.55	0.58	41.58	1521.02	1.34
			(0.05)	(0.06)	(0.06)	(0.05)	(0.06)	(44.16)	(2956.5)	(0.71)
		GM-PAM	0.55	0.55	0.55	0.55	0.58	45.42	1651.47	1.28
			(0.05)	(0.06)	(0.06)	(0.05)	(0.06)	(45.04)	(3051.22)	(0.7)
	0.6	PAM	0.54	0.65	0.44	0.53	0.56	76.58	4244.76	0.81
			(0.04)	(0.06)	(0.09)	(0.04)	(0.05)	(36.56)	(3526.27)	(0.81)
		GM-PAM	0.54	0.65	0.43	0.53	0.56	77.71	4500.95	0.76
			(0.04)	(0.06)	(0.09)	(0.04)	(0.05)	(35.82)	(3609.07)	(0.79)
	0.7	PAM	0.53	0.75	0.31	0.48	0.55	91.95	5468.92	0.51
			(0.02)	(0.05)	(0.07)	(0.04)	(0.03)	(22.71)	(2926.65)	(0.47)
		GM-PAM	0.53	0.75	0.31	0.48	0.55	93.32	5759.93	0.46
			(0.02)	(0.05)	(0.06)	(0.04)	(0.03)	(20.39)	(2888.1)	(0.43)
	0.8	PAM	0.52	0.85	0.19	0.4	0.55	95.17	5993.06	0.48
			(0.02)	(0.04)	(0.05)	(0.05)	(0.03)	(15.11)	(2564.83)	(0.35)
		GM-PAM	0.52	0.85	0.19	0.4	0.55	95.42	6203.27	0.46
			(0.02)	(0.04)	(0.05)	(0.05)	(0.03)	(15.07)	(2576.17)	(0.35)
	0.9	PAM	0.51	0.95	0.06	0.24	0.53	82.72	6012.49	0.69
			(0.01)	(0.02)	(0.04)	(0.06)	(0.03)	(25.81)	(2688.55)	(0.54)
		GM-PAM	0.51	0.95	0.06	0.24	0.53	83.95	6161.64	0.65
			(0.01)	(0.02)	(0.03)	(0.06)	(0.03)	(25.24)	(2631.05)	(0.51)
1	0.5	PAM	0.69	0.69	0.69	0.69	0.77	46.46	479.55	2.16
			(0.03)	(0.05)	(0.05)	(0.03)	(0.04)	(42.87)	(1525.94)	(0.94)
		GM-PAM	0.69	0.69	0.7	0.69	0.76	50.47	513.59	2.1
			(0.03)	(0.05)	(0.05)	(0.03)	(0.04)	(42.49)	(1532.66)	(0.94)
	0.6	PAM	0.68	0.73	0.63	0.67	0.74	70.97	1690.36	1.72
			(0.04)	(0.04)	(0.08)	(0.04)	(0.05)	(42.27)	(2251.51)	(1.34)
		GM-PAM	0.68	0.73	0.62	0.67	0.74	73.08	1808.83	1.67
			(0.04)	(0.04)	(0.09)	(0.04)	(0.05)	(41.41)	(2341.65)	(1.33)
	0.7	PAM	0.63	0.79	0.48	0.61	0.7	91.13	3848.5	0.96
			(0.05)	(0.04)	(0.12)	(0.07)	(0.06)	(26.49)	(3113.97)	(1.02)
		GM-PAM	0.63	0.79	0.47	0.6	0.69	93.71	4157.24	0.87
			(0.05)	(0.04)	(0.12)	(0.07)	(0.06)	(22.79)	(3103.25)	(0.96)
	0.8	PAM	0.6	0.86	0.33	0.52	0.67	97.33	5429.52	0.7
			(0.05)	(0.04)	(0.14)	(0.09)	(0.06)	(15.13)	(3103.96)	(0.85)
		GM-PAM	0.59	0.87	0.31	0.51	0.67	97.38	5797.09	0.63
			(0.05)	(0.04)	(0.13)	(0.09)	(0.06)	(15.12)	(3019.79)	(0.83)
	0.9	PAM	0.54	0.95	0.12	0.32	0.62	96.17	5651.55	0.81
			(0.03)	(0.03)	(0.09)	(0.09)	(0.06)	(13.01)	(2577.79)	(0.78)
		GM-PAM	0.53	0.95	0.12	0.32	0.62	96.17	5789.34	0.78
			(0.03)	(0.03)	(0.09)	(0.09)	(0.05)	(13.01)	(2616.25)	(0.78)

Figure 1: **Classification results obtained after over-sampling the training set.** The figure shows PA for class 1 (PA_1) and PA for class 2 (PA_2) for different levels of class-imbalance (k_1) in the training set containing 100 samples and difference between the classes (left panel: $\mu_2 = 0$, middle panel: $\mu_2 = 0.5$, right panel: $\mu_2 = 1$). The simulation setting were the same as presented in the table above. See text for more details.

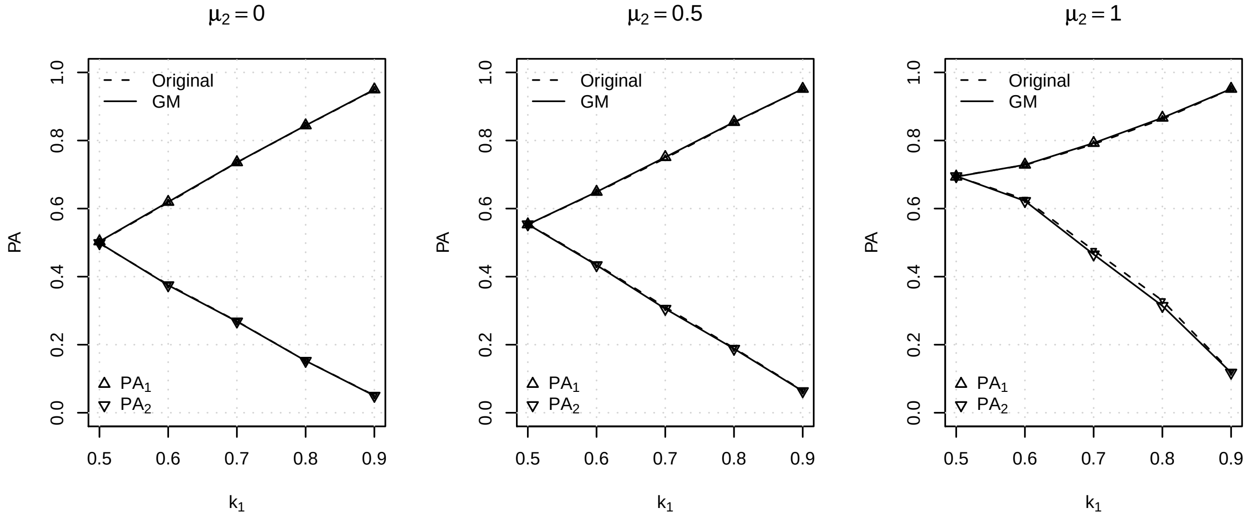

Supplement: Additional file 8 — Classification results for the over-sampled training set for PAM and GM-PAM. In the additional file we show the simulation results obtained by training PAM and GM-PAM on over-sampled training sets obtained by replicating randomly selected samples from the minority class in order to obtain the class-balanced training set. Simulation settings were the same as presented in the Additional file 4. See text for more details. [file 1471-2105-14-64-S8.pdf]
