# Supplementary material for: Improved shrunken centroid classifiers for high-dimensional class-imbalanced data
Source: BMC Bioinformatics. 2013 Feb 23;14:64. doi: 10.1186/1471-2105-14-64 (PMC3687811; doi:10.1186/1471-2105-14-64)
Supplement: Additional file 9 — Classification results for the three class scenario. In the additional file we show the same information as in Additional file 4 for the three class scenario. In Tables 1 and 2 we report results for the the class-balanced scenario (n1=n2=n3=100) and a large number of variables (p=5000) under the null and the alternative hypothesis, respectively. Table 3 reports the results for the class-imbalanced scenario (n1=n3=100, n2=20) under the null hypothesis and Table 4 reports the result under the alternative hypothesis. Results for smaller number of variables are in Table 5. [file 1471-2105-14-64-S9.pdf]

Table 1: **Multi-class classification results for the class-balanced scenario ($n_1 = n_2 = n_3 = 100$) and a large number of variables ($p = 5000$) under the null hypothesis**. In the table we report the average number and standard deviation (in brackets) of active irrelevant variables (# non-info), optimal threshold parameter (λ^*), class-specific PAs (PA_1, PA_2, PA_3) and g-means for different methods. There was no difference between the classes ($\mu_1 = 0$).

Method	λ^*	# non-info	PA_1	PA_2	PA_3	g-means
PAM	1.23	1060.67	0.33	0.33	0.33	0.31
	(0.63)	(1719.31)	(0.11)	(0.12)	(0.12)	(0.03)
GM-PAM	1.08	1436.99	0.33	0.33	0.34	0.32
	(0.66)	(1880.97)	(0.07)	(0.08)	(0.08)	(0.02)
ALP	3.77	962.82	0.37	0.33	0.3	0.26
	(2.6)	(1600.19)	(0.09)	(0.13)	(0.14)	(0.13)
GM-ALP	2.93	1498.77	0.35	0.33	0.32	0.29
	(2.67)	(1904.14)	(0.09)	(0.1)	(0.12)	(0.11)
AHP	3.56	1203.8	0.37	0.35	0.27	0.24
	(2.65)	(1846.18)	(0.09)	(0.12)	(0.15)	(0.14)
GM-AHP	3	1648.55	0.35	0.33	0.31	0.28
	(2.87)	(1999.59)	(0.07)	(0.1)	(0.12)	(0.11)

Table 2: **Multi-class classification results for the class-balanced scenario ($n_1 = n_2 = n_3 = 100$) and a large number of variables ($p = 5000$) under the alternative hypothesis**. In the table we report the average number and standard deviation (in brackets) of active relevant variables (out of 100; # info) and active irrelevant variables (# non-info), optimal threshold parameter (λ^*), class-specific PAs (PA_1, PA_2, PA_3) and g-means for different methods. The difference between the classes was moderate ($\mu_1 = 1$).

Method	λ^*	# info	# non-info	PA_1	PA_2	PA_3	g-means
PAM	1.98	99	388.3	0.77	0.29	0.76	0.54
	(1.27)	(8.81)	(791.72)	(0.05)	(0.09)	(0.05)	(0.06)
GM-PAM	0.85	100	1310.99	0.73	0.36	0.73	0.58
	(0.41)	(0.00)	(1368.33)	(0.04)	(0.04)	(0.04)	(0.02)
ALP	19.97	98.69	28.39	0.76	0.32	0.76	0.55
	(17.43)	(7.31)	(56.62)	(0.05)	(0.10)	(0.05)	(0.06)
GM-ALP	5.87	100	298.54	0.72	0.39	0.72	0.59
	(4.53)	(0.00)	(1063.33)	(0.04)	(0.03)	(0.04)	(0.02)
AHP	18.24	99.17	27.37	0.75	0.33	0.75	0.56
	(14.72)	(5.87)	(82.63)	(0.04)	(0.09)	(0.05)	(0.05)
GM-AHP	5.69	100	450.2	0.72	0.39	0.71	0.59
	(4.52)	(0.00)	(1321.77)	(0.04)	(0.04)	(0.04)	(0.02)

Table 3: **Multi-class classification results for the class-imbalanced scenario ($n_1 = n_3 = 100$, $n_2 = 20$) under the null hypothesis**. In the table we report the average number and standard deviation (in brackets) of active irrelevant variables (out of $p = 1000$ or 5000; # non-info), optimal threshold parameter (λ^*), class-specific PAs (PA_1, PA_2, PA_3) and g-means for different methods. There was no difference between the classes ($\mu_1 = 0$).

p	Method	λ^*	# non-info	PA_1	PA_2	PA_3	g-means
1000	PAM	0.97	330.17	0.38	0.26	0.37	0.3
		(0.66)	(444.16)	(0.12)	(0.11)	(0.12)	(0.05)
	GM-PAM	0.96	313.43	0.34	0.33	0.33	0.32
		(0.6)	(404.05)	(0.09)	(0.11)	(0.1)	(0.03)
	ALP	0.91	646.53	0.36	0.31	0.34	0.31
		(1.44)	(386.93)	(0.1)	(0.08)	(0.1)	(0.05)
	GM-ALP	1.78	457.27	0.34	0.35	0.31	0.29
		(2.00)	(404.72)	(0.13)	(0.08)	(0.13)	(0.08)
	AHP	0.93	643.62	0.33	0.32	0.34	0.31
		(1.61)	(399.6)	(0.11)	(0.08)	(0.1)	(0.05)
	GM-AHP	1.53	520.89	0.34	0.34	0.31	0.29
		(2.05)	(421.31)	(0.11)	(0.08)	(0.12)	(0.08)
5000	PAM	0.64	3101.86	0.4	0.2	0.41	0.3
		(0.79)	(2310.45)	(0.1)	(0.08)	(0.09)	(0.03)
	GM-PAM	1.1	1304.05	0.34	0.31	0.35	0.32
		(0.63)	(1758.05)	(0.08)	(0.1)	(0.07)	(0.02)
	ALP	0.85	3596.27	0.4	0.21	0.38	0.3
		(1.77)	(1886.72)	(0.07)	(0.09)	(0.08)	(0.05)
	GM-ALP	2.64	1340.91	0.34	0.31	0.34	0.3
		(2.33)	(1535.67)	(0.12)	(0.1)	(0.11)	(0.06)
	AHP	0.5	3748.31	0.4	0.21	0.39	0.31
		(0.93)	(1670.28)	(0.07)	(0.07)	(0.06)	(0.02)
	GM-AHP	2.85	1336.29	0.35	0.32	0.33	0.29
		(2.6)	(1626.17)	(0.14)	(0.1)	(0.14)	(0.07)

Table 4: **Multi-class classification results for the class-imbalanced scenario ($n_1 = n_3 = 100$, $n_2 = 20$) under the alternative hypothesis**. In the table we report the average number and standard deviation (in brackets) of active relevant variables (out of 100; # info) and active irrelevant variables (out of 900 or 4900; # non-info), optimal threshold parameter (λ^*), class-specific PAs (PA_1, PA_2, PA_3) and g-means for different methods. The difference between the classes was moderate ($\mu_1 = 0.5$).

p	Method	λ^*	# info	# non-info	PA_1	PA_2	PA_3	g-means
1000	PAM	3.34	56.03	0	0.7	0.03	0.7	0.24
		(0.51)	(31.53)	(0.04)	(0.03)	(0.01)	(0.03)	(0.03)
	GM-PAM	0.94	91.56	406.51	0.59	0.2	0.59	0.4
		(1.06)	(27.16)	(355.76)	(0.09)	(0.07)	(0.09)	(0.04)
	ALP	9.71	99.97	15.74	0.65	0.12	0.66	0.36
		(5.52)	(0.17)	(65.57)	(0.06)	(0.05)	(0.06)	(0.04)
	GM-ALP	5.12	95.06	265.16	0.57	0.22	0.58	0.4
		(7.25)	(21.18)	(373.33)	(0.14)	(0.07)	(0.14)	(0.05)
	AHP	10.46	99.93	17.59	0.66	0.11	0.66	0.36
		(5.00)	(0.70)	(98)	(0.05)	(0.04)	(0.05)	(0.03)
	GM-AHP	5.96	91.86	284.23	0.58	0.22	0.57	0.39
		(9.07)	(26.37)	(370.9)	(0.13)	(0.09)	(0.14)	(0.07)
5000	PAM	3.35	52.75	0.76	0.7	0.03	0.7	0.24
		(0.49)	(32.41)	(14.55)	(0.04)	(0.02)	(0.03)	(0.03)
	GM-PAM	1.25	88.21	1364.98	0.6	0.18	0.59	0.39
		(1.11)	(31.22)	(1489.87)	(0.09)	(0.07)	(0.09)	(0.05)
	ALP	10.59	99.92	247.38	0.66	0.11	0.65	0.35
		(5.5)	(0.37)	(990.3)	(0.05)	(0.05)	(0.08)	(0.04)
	GM-ALP	6.42	92.16	902.72	0.59	0.2	0.58	0.38
		(8.22)	(24.18)	(1403.05)	(0.13)	(0.1)	(0.15)	(0.06)
	AHP	9.49	99.84	243.91	0.65	0.12	0.65	0.35
		(5.49)	(1.12)	(971.89)	(0.06)	(0.06)	(0.08)	(0.05)
	GM-AHP	5.88	92.39	933.81	0.58	0.21	0.58	0.38
		(7.89)	(24.91)	(1408.52)	(0.13)	(0.09)	(0.13)	(0.06)

Table 5: **Multi-class classification results for the class-imbalanced scenario ($n_1 = n_3 = 100$, $n_2 = 20$) and a smaller number of variables ($p = 1000$)**. In the table we report the average number and standard deviation (in brackets) of active relevant variables (out of 100; # info) and active irrelevant variables (# non-info), optimal threshold parameter (λ^*), class-specific PAs (PA_1, PA_2, PA_3) and g-means for different methods. The difference between the classes was moderate ($\mu_1 = 1$).

Method	λ^*	# info	# non-info	PA_1	PA_2	PA_3	g-means
PAM	6.55	61.12	0	0.86	0.04	0.86	0.29
	(0.62)	(32.99)	(0)	(0.02)	(0.02)	(0.02)	(0.05)
GM-PAM	1.07	100	285.29	0.74	0.33	0.74	0.57
	(0.89)	(0)	(328.57)	(0.04)	(0.05)	(0.04)	(0.03)
ALP	61.24	100	0	0.83	0.14	0.83	0.45
	(13.46)	(0)	(0)	(0.04)	(0.05)	(0.04)	(0.05)
GM-ALP	10.36	100	172.2	0.73	0.35	0.73	0.57
	(9.55)	(0)	(352.25)	(0.05)	(0.06)	(0.05)	(0.03)
AHP	60.31	100	0	0.81	0.15	0.82	0.46
	(15.26)	(0)	(0)	(0.04)	(0.05)	(0.04)	(0.05)
GM-AHP	10.08	100	217.3	0.73	0.35	0.73	0.57
	(11.94)	(0)	(383.19)	(0.05)	(0.06)	(0.05)	(0.03)
