# Supplementary material for: Improved shrunken centroid classifiers for high-dimensional class-imbalanced data
Source: BMC Bioinformatics. 2013 Feb 23;14:64. doi: 10.1186/1471-2105-14-64 (PMC3687811; doi:10.1186/1471-2105-14-64)
Supplement: Additional file 11 — Classification results for the gene expression data sets. Results on the Sotiriou (2003) data set for classification of ER and Grade of the tumor (Table 1) and on the Ivshina data set for the multi class classification task (Table 2). [file 1471-2105-14-64-S11.pdf]

Table 1: **Results on the Sotiriou (2003) data set for classification of ER and Grade of the tumor.** In the table we report overall PA (PA), class specific PAs (PA_1, PA_2), g-means, AUC, number of active genes (# gene) and the optimal threshold parameter (λ^*), for different number of ER- or Grade 1 or 2 samples in the training set (n_1). The number of ER+ and Grade 3 samples was kept fixed at 10.

Task	Method	n_1	PA	PA_1	PA_2	g-means	AUC	# genes	λ^*
ER	PAM	10	0.831	0.833	0.829	0.827	0.893	267	2.900
	GM-PAM	10	0.831	0.833	0.827	0.827	0.893	322	2.852
	PAM	20	0.832	0.830	0.836	0.830	0.893	933	2.474
	GM-PAM	20	0.830	0.824	0.843	0.831	0.895	693	2.598
	PAM	30	0.829	0.844	0.808	0.822	0.883	1892	1.799
	GM-PAM	30	0.832	0.831	0.835	0.830	0.896	944	2.266
	PAM	40	0.827	0.856	0.796	0.822	0.875	3132	1.259
	GM-PAM	40	0.831	0.828	0.834	0.828	0.894	1287	2.041
	PAM	50	0.808	0.889	0.757	0.817	0.865	4525	0.703
	GM-PAM	50	0.834	0.846	0.827	0.833	0.896	1527	1.791
	ALP	10	0.829	0.834	0.819	0.823	0.877	70	21.910
	GM-ALP	10	0.829	0.834	0.817	0.822	0.877	90	21.763
	ALP	20	0.848	0.850	0.844	0.845	0.898	333	16.498
	GM-ALP	20	0.846	0.847	0.844	0.844	0.897	342	16.722
	ALP	30	0.845	0.856	0.830	0.841	0.903	1106	11.303
	GM-ALP	30	0.844	0.848	0.838	0.841	0.900	572	13.434
	ALP	40	0.834	0.868	0.798	0.829	0.905	1864	9.099
	GM-ALP	40	0.843	0.858	0.829	0.841	0.907	610	11.590
	ALP	50	0.824	0.883	0.786	0.830	0.906	2755	6.923
	GM-ALP	50	0.840	0.860	0.827	0.841	0.903	594	12.710
	AHP	10	0.854	0.860	0.839	0.847	0.902	212	32.995
	GM-AHP	10	0.854	0.860	0.839	0.847	0.902	212	32.608
	AHP	20	0.863	0.880	0.831	0.853	0.914	212	35.653
	GM-AHP	20	0.862	0.879	0.829	0.851	0.914	272	34.800
	AHP	30	0.860	0.899	0.804	0.848	0.908	399	38.455
	GM-AHP	30	0.861	0.899	0.806	0.849	0.909	324	40.269
	AHP	40	0.859	0.911	0.806	0.855	0.910	381	38.345
	GM-AHP	40	0.860	0.910	0.807	0.856	0.911	351	38.746
	AHP	50	0.833	0.912	0.784	0.842	0.908	336	37.445
	GM-AHP	50	0.836	0.914	0.787	0.845	0.909	303	38.261
Grade	PAM	10	0.599	0.622	0.572	0.585	0.629	508	1.959
	GM-PAM	10	0.600	0.623	0.571	0.586	0.632	536	1.893
	PAM	20	0.616	0.673	0.562	0.607	0.665	1266	1.662
	GM-PAM	20	0.615	0.665	0.566	0.605	0.662	761	1.835
	PAM	30	0.607	0.720	0.529	0.608	0.667	2945	1.110
	GM-PAM	30	0.616	0.673	0.576	0.615	0.667	1014	1.713
	PAM	40	0.566	0.771	0.485	0.601	0.660	4866	0.527
	GM-PAM	40	0.595	0.686	0.559	0.608	0.661	1440	1.566
	ALP	10	0.611	0.637	0.579	0.598	0.656	525	8.190
	GM-ALP	10	0.618	0.647	0.582	0.605	0.661	567	7.471
	ALP	20	0.621	0.690	0.554	0.608	0.662	932	7.907
	GM-ALP	20	0.620	0.675	0.566	0.610	0.662	551	8.224
	ALP	30	0.608	0.740	0.517	0.609	0.684	2219	5.435
	GM-ALP	30	0.610	0.699	0.549	0.613	0.670	871	7.414
	ALP	40	0.571	0.757	0.497	0.603	0.698	3519	3.196
	GM-ALP	40	0.597	0.696	0.558	0.615	0.682	739	6.802
	AHP	10	0.589	0.607	0.566	0.579	0.614	438	13.207
	GM-AHP	10	0.599	0.607	0.588	0.591	0.627	446	11.695
	AHP	20	0.619	0.731	0.510	0.602	0.673	445	10.074
	GM-AHP	20	0.618	0.728	0.510	0.601	0.671	434	10.323
	AHP	30	0.593	0.774	0.470	0.597	0.679	463	9.875
	GM-AHP	30	0.591	0.759	0.476	0.595	0.669	309	10.941
	AHP	40	0.560	0.795	0.466	0.603	0.700	865	9.156
	GM-AHP	40	0.554	0.780	0.463	0.593	0.688	673	10.521

Table 2: **Performance of the classifiers on the Ivshina data set for the multi class classification task.** The table shows overall PA (PA) class specific PAs (PA_1, PA_2, PA_3), g-means, optimal threshold parameter (λ^*) and the number of active genes (# genes) for the prediction of Grade of the tumor. Optimal threshold parameters were estimated with 5-fold CV and the accuracy measures with LOOCV.

Method	λ^*	# genes	PA	PA_1	PA_2	PA_3	g-means
PAM	0.00	22283	0.62	0.76	0.50	0.82	0.68
GM-PAM	0.57	17776	0.62	0.76	0.49	0.84	0.68
ALP	0.00	22283	0.62	0.75	0.51	0.82	0.68
GM-ALP	0.00	22283	0.61	0.75	0.49	0.82	0.67
AHP	0.00	22283	0.62	0.74	0.51	0.82	0.67
GM-AHP	0.00	22283	0.62	0.74	0.51	0.82	0.67
